# Mediation Role of Gut Microbiota in the Causal Relationship Between m6A Regulatory Genes and Metabolic Dysfunction-Associated Steatotic Liver Disease: A Mendelian Randomization Study

**DOI:** 10.3390/biomedicines14030630

**Published:** 2026-03-11

**Authors:** Dongmei Qiu, Liwei Suo, Tao Wei, Zhenwei Lu, Qixin Weng, Jianxing Xiao, Xinchi Wang, Qinyu Xu, Jingtong Wu

**Affiliations:** 1 Department of Gastroenterology, Clinical Research Center for Gut Microbiota and Digestive Diseases of Fujian Province, The National Key Clinical Specialty, Zhongshan Hospital of Xiamen University, School of Medicine, Xiamen University, Xiamen 361004, China; 24520240157796@stu.xmu.edu.cn (D.Q.); slw18334162591@163.com (L.S.); 2 Department of Digestive Disease, Institute for Microbial Ecology, School of Medicine, Xiamen University, Xiamen 361004, China; 21620231153609@stu.xmu.edu.cn (T.W.); 13024830705@163.com (Z.L.); wengqx77@163.com (Q.W.); 18334885384@163.com (J.X.); wxc137046736@126.com (X.W.)

**Keywords:** Mendelian randomization, m6A, gut microbiota, MASLD, mediation analysis

## Abstract

**Background**: Metabolic dysfunction-associated steatotic liver disease (MASLD) is a globally prevalent condition with a complex pathogenesis. While both m6A RNA methylation regulators and gut microbiota have been independently implicated in MASLD, their potential causal interplay remains unexplored. This study aimed to investigate the causal relationships among m6A regulatory genes, gut microbiota, and MASLD, and to assess the mediating role of gut microbiota. **Methods**: We performed a two-sample Mendelian randomization (MR) analysis using publicly available genome-wide association study (GWAS) data. Genetic instruments for m6A regulators were derived from blood expression quantitative trait loci (eQTL) data. Gut microbiota and MASLD data were obtained from large-scale metagenomic and disease GWAS, respectively. The inverse-variance weighted method was the primary analysis, supplemented by sensitivity and mediation analyses to evaluate potential mediating pathways. **Results**: Genetically predicted levels of four m6A regulators showed significant causal associations with MASLD risk: ALKBH3 increased risk (OR = 1.17), whereas ALKBH5 (OR = 0.89), CBLL1 (OR = 0.76), and RBM15B (OR = 0.83) were protective. Nineteen gut microbial taxa were causally linked to MASLD. Among these, seven taxa were influenced by the four identified m6A genes. Although no mediation effects reached strict statistical significance, the pathway from ALKBH5 to MASLD via Parabacteroides abundance showed a suggestive indirect effect accounting for 21.9% of the total effect (*p* = 0.068). Given the limited statistical power of mediation analyses in MR settings, this observation should be interpreted with caution and requires validation in larger, well-powered studies. **Conclusions**: This MR study provides genetic evidence supporting causal roles of specific m6A regulators in MASLD and suggests that gut microbiota may partially mediate these relationships. The findings highlight a potential “m6A–gut microbiota–liver” axis in MASLD pathogenesis.

## 1. Introduction

MASLD, which is known as the former NAFLD (Nonalcoholic Fatty Liver Disease), is the most common chronic liver disease globally. Its prevalence has risen from 25.3% (1990–2006) to 38.0% (2016–2019) [1], paralleling the global epidemics of obesity and type 2 diabetes. The pathological features of MASLD are the excessive deposition of triglycerides in the liver. The subsequent steatosis and fibrotic changes in the liver then lead to the progressive form, metabolic dysfunction-associated steatohepatitis (MASH) [2,3].

N^6^-methyladenosine (m6A), the most prevalent internal chemical modification in eukaryotic mRNA, is catalyzed by the METTL3-METTL14-WTAP complex and can be dynamically erased by demethylases such as FTO and ALKBH5 [4,5]. m6A plays crucial roles in various biological processes including stem cell differentiation, embryonic development, neural function, and immune responses by influencing RNA splicing, nuclear export, stability, and translation [6]. As a key mechanism of post-transcriptional regulation, m6A has become a central focus in epitranscriptomics research.

The gut microbiota has been implicated in the pathogenesis of MASLD. Studies have demonstrated that patients with MASLD exhibit reduced microbial diversity and altered gut microbiota composition [7,8]. The gut microbiota plays a crucial role in the development of MASLD through the “gut–liver axis”—a bidirectional communication network that integrates anatomical, immunological, and metabolic signals between the intestinal lumen and the liver [9]. Dysbiosis disrupts the intestinal barrier, increasing permeability and allowing microbial products, such as lipopolysaccharides (LPS), short-chain fatty acids (SCFAs), and secondary bile acids, to translocate into the portal circulation [9]. In the liver, these metabolites activate pro-inflammatory pathways, promoting hepatic steatosis, inflammation, and fibrosis [7].

However, current research has largely been confined to exploring the associations of m6A modification and gut microbiota with MASLD separately. While this evidence suggests that both host epitranscriptomic regulation and microbial composition are implicated in MASLD, whether gut microbiota mediates the causal pathway from m6A regulatory genes to MASLD risk remains entirely unexplored. Therefore, this study aims to employ Mendelian randomization to systematically evaluate the causal relationships between m6A regulatory genes, gut microbiota, and MASLD, with highlighting the potential mediating role of gut microbiota, thereby providing new insights into the “epitranscriptome-microbiota axis” in MASLD pathogenesis [10].

## 2. Materials and Methods

### 2.1. Data Sources

This two-sample MR analysis utilized publicly available GWAS summary data.

m6A Regulatory Genes: Genetic instruments for m6A regulatory genes were constructed using expression quantitative trait loci (eQTL) and protein quantitative trait loci (pQTL) data.

- Compilation of Gene List: We first compiled a list of 46 known m6A regulators based on a comprehensive literature review [11,12,13,14,15,16,17,18].
- eQTL data: eQTL summary statistics were derived from the eQTLGen (https://www.eqtlgen.org/cis-eqtls.html, accessed on 17 September 2025) [19]. We filtered instrumental variables using the following criteria: *p*-value < 5 × 10^−8^, clumping window: 10,000 kb, r^2^ < 0.1, resulting in 15,695 genes with significant cis-eQTLs. By intersecting these genes with the aforementioned 46 m6A genes (Supplementary Figure S1), we identified 24 m6A genes with significant cis-eQTLs: ALKBH1, ALKBH3, ALKBH5, CBLL1, CEBPZ, ELAVL1, FTO, IGF2BP2, IGF2BP3, KIAA1429, METTL14, METTL16, METTL3, METTL5, METTL7A, RBM15, RBM15B, SMAD2, SMAD3, WTAP, YTHDC2, YTHDF2, YTHDF3, ZC3H13.
- pQTL data: pQTL data were obtained from the deCODE database (https://www.decode.com/summarydata/, accessed on 17 September 2025) [20]. A total of 4907 raw files from an Icelandic population were downloaded. We extracted cis-pQTLs using the following criteria: SNPs located on the same chromosome as the gene and within ±1 Mb of the gene region, yielding 4706 cis-pQTL files. Further filtering was performed with *p*-value < 5 × 10^−8^, clumping window: 10,000 kb, r^2^ < 0.1, resulting in 1615 cis-pQTL data files. Intersection with 46 m6A genes revealed only ALKBH3 possessed a significant cis-pQTL (Supplementary Figure S1).

Gut Microbiota: Genetic instruments for gut were sourced from a large-scale GWAS published by Qin et al. [21]. This study included metagenomic sequencing data from 5959 individuals, aiming to identify genetic loci influencing human gut microbiome composition and function. Given that the stringent genome-wide significance threshold (*p* < 5 × 10^−8^) would exclude >80% of candidate SNPs and compromise statistical power in Mendelian randomization (MR), we selected single nucleotide polymorphisms (SNPs) as instruments based on a pre-set significance threshold (*p* < 5.0 × 10^−6^) and clumped them for linkage disequilibrium (r^2^ < 0.001 within a 10,000kb distance) to obtain independent instruments. Ultimately, a total of 473 microbial feature-associated instruments were obtained for subsequent analysis.

MASLD: The outcome GWAS summary-level data were obtained from the GWAS Catalog (https://www.ebi.ac.uk/gwas/, accessed on 18 September 2025). This dataset (GCST90091033) contained 778,614 European individuals, including 8434 cases and 770,180 controls of European ancestry [22]. This meta-analysis included four electronic health record-based cohorts: eMERGE, UK Biobank, Estonian Biobank, and FinnGen [22]. NAFLD diagnosis was defined using harmonized ICD codes across cohorts (ICD-9: 571.5, 571.8, 571.9; ICD-10: K75.81, K76.0, K76.9, and additional codes specific to each cohort). Individuals with other liver diseases (e.g., alcoholic liver disease, viral hepatitis) were excluded following AASLD guidelines. The original GWAS implemented stringent quality control measures, including population stratification adjustment using SAIGE and sensitivity analyses, to minimize potential bias from diagnostic heterogeneity.

It is important to note that different LD pruning thresholds were used for the two exposures due to differences in instrument availability. For gut microbiota, a stringent threshold (r^2^ < 0.001) was applied to ensure SNP independence, which is standard practice in microbiome MR studies. For m6A eQTLs, the same strict threshold removed most cis-eQTLs, resulting in insufficient instruments and potential weak instrument bias. To balance instrument strength with independence, we adopted a more lenient threshold (r^2^ < 0.1), consistent with previous MR studies [23].

All datasets included in our analysis were publicly available with existing ethical approvals from their original sources. As this study utilized only summary-level data, no additional ethical approval was required.

### 2.2. Study Design

**Figure 1:**
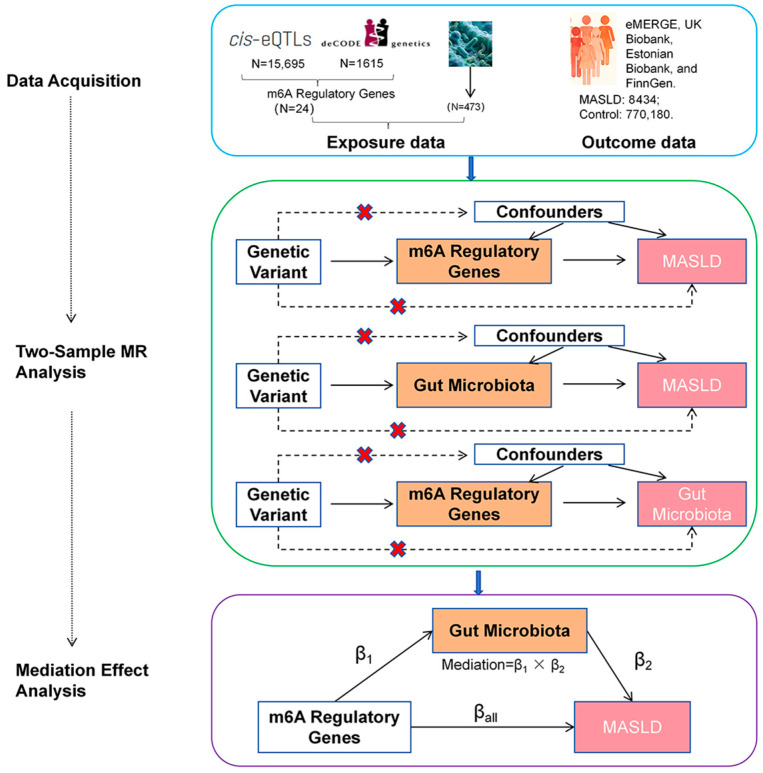
Study design flowchart.

This study employed a two-step MR analysis to investigate the causal relationship between m6A regulatory genes and MASLD and to investigate whether gut microbiota act as mediators [10]. The total effect of m6A genes on MASLD can be divided into a direct effect and an indirect effect mediated by gut microbiota. In step 1, we performed two-sample MR using summary-level GWAS data to estimate the total effect of the 24 m6A regulatory genes on MASLD. In step 2, the mediation analysis was applied to evaluate the mediating role of the 473 gut microbiota in the causal pathway from m6A genes to MASLD and to quantify the indirect effect (Figure 1).

This flowchart illustrates the overall study design and the four main analytical steps: (1) two-sample Mendelian randomization (MR) analysis of the causal effects of m6A regulatory genes on MASLD; (2) two-sample MR analysis of the causal effects of gut microbiota on MASLD; (3) two-sample MR analysis of the causal effects of m6A regulatory genes on gut microbiota; and (4) two-step mediation analysis to assess the indirect effects of gut microbiota in the m6A-MASLD relationship. Data sources and sample sizes for each step are indicated.

This MR study strictly followed the STROBE-MR guidelines and was based on three key MR assumptions: (1) Instrumental variables (IVs) are strongly associated with the exposure; (2) IVs are independent of confounding factors affecting the exposure-outcome relationship; (3) IVs influence the outcome (MASLD) only through the exposure, not via other direct or indirect pathways [24].

### 2.3. Statistical Analysis

#### 2.3.1. Two-Sample Mendelian Randomization Analysis

This study utilized the TwoSampleMR package to conduct a two-sample Mendelian randomization (MR) analysis. Cis-eQTL, cis-pQTL data corresponding to m6A regulatory genes, and gut microbiota were employed as exposures, with MASLD as the outcome. To minimize bias introduced by weak instrumental variables (IVs), single nucleotide polymorphisms (SNPs) with F-statistics less than 10 were excluded. Additionally, we applied Steiger filtering to test the directionality of instrumental variables, excluding SNPs that explained more variance in the outcome than in the exposure. Only genes containing at least one SNP that passed all quality control steps were retained for subsequent analyses.

#### 2.3.2. Mediation Analysis

A two-step MR framework was used for mediation analysis to assess the potential mediating effect of gut microbiota in the m6A regulatory genes–MASLD relationship. In step 1, we used two-sample MR to estimate the total effect of each m6A gene on MASLD, denoted as βall. In step 2, we employed mediation MR analysis to quantify the mediating effect of gut microbiota in the causal pathway from m6A genes to MASLD. The specific steps were as follows: Assess the causal effect of gut microbiota on MASLD, denoted as β_2_. Assess the causal effect of the m6A gene on gut microbiota, denoted as β_1_. The indirect effect (β12) of the m6A gene on MASLD was calculated as the product β_1_ × β_2_. The direct effect was obtained by subtracting the indirect effect from the total effect (βall—β_1_ × β_2_). The proportion mediated was calculated as (β_1_ × β_2_)/βall. 95% confidence intervals (CIs) for all effects were calculated using the propagation of error method [10].

#### 2.3.3. Sensitivity Analysis

MR analyses were performed using multiple methods, with results expressed as odds ratios (ORs) and regression coefficients (β values). The inverse-variance weighted (IVW) method with random effects was used as the primary analysis method [10]. To verify the reliability of IVW estimates, we performed a series of sensitivity analyses, including the MR-Egger, weighted median, simple mode, and weighted mode methods. Cochran’s Q test was used to evaluate heterogeneity among genetic variants, with *p* > 0.05 indicating no significant heterogeneity [25]. The intercept term from MR-Egger regression was employed to assess horizontal pleiotropy, with *p* > 0.05 suggesting no evidence of pleiotropy [26]. The MR-Pleiotropy Residual Sum and Outlier (MR-PRESSO) method was applied to detect and correct for potential outliers influenced by horizontal pleiotropy. A Global Test *p* > 0.05 indicated the absence of horizontal pleiotropic outliers. Leave-one-out analysis was performed by sequentially excluding each instrumental variable to evaluate its impact on the overall effect estimate. Furthermore, scatter plots and funnel plots were generated for visualization. All MR analyses were performed using R software (version 4.4.1) with the “TwoSampleMR” package (version 0.6.11) [27].

## 3. Results

### 3.1. Causal Effects of m6A Regulatory Genes on MASLD

**Figure 2:**
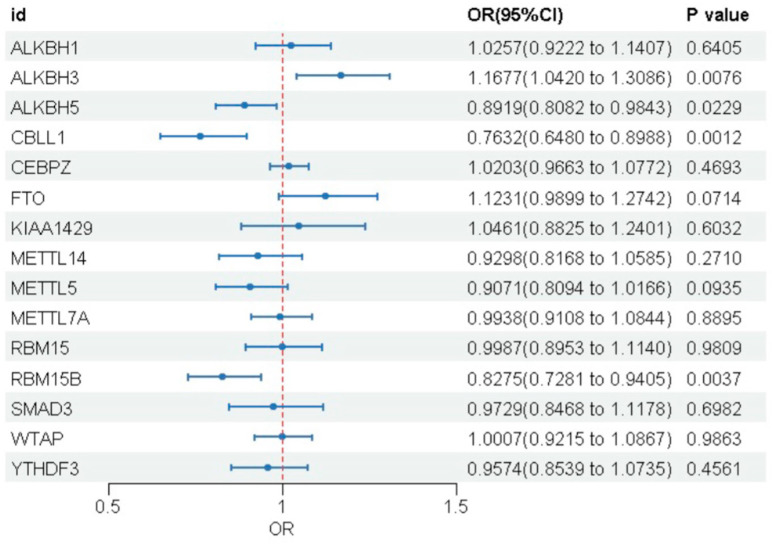
Forest plot showing the causal associations between m6A regulatory gene expression and MASLD risk.

To investigate the association between the 24 m6A genes significantly associated with eQTLs and MASLD, we performed a two-sample Mendelian randomization analysis, using the IVW method as the primary approach. After excluding SNPs with F-statistic ≤ 10 and applying Steiger filtering (Supplementary Table S1), we found associations between 15 m6A genes and MASLD risk; however, only 4 genes demonstrated statistically significant causality (*p* < 0.05) (Figure 2 and Table 1). Genetically predicted higher expression of ALKBH3 was associated with an increased risk of MASLD (OR = 1.168), while elevated expression of ALKBH5 (OR = 0.892), CBLL1 (OR = 0.763), and RBM15B (OR = 0.828) was associated with a decreased risk. Among these, CBLL1 showed the strongest protective effect, corresponding to a 23.7% reduction in MASLD risk. To verify the reliability of our findings, we performed systematic sensitivity analyses. Results from 4 alternative MR methods were consistent with the IVW estimates, further supporting the stability of the causal associations (Supplementary Table S2). Cochran’s Q test showed no significant heterogeneity (all *p* > 0.05), supporting the robustness of the causal estimates (Supplementary Table S3). MR–Egger intercept analysis revealed no evidence of horizontal pleiotropy (all *p* > 0.05) (Supplementary Figure S2). The MR-PRESSO global test detected no outliers, and leave-one-out analysis confirmed that the estimates were not driven by any single SNP (Supplementary Figure S3). Scatter and funnel plots were also generated (Supplementary Figures S4 and S5). In contrast to the transcript-level findings, no causal link was observed at the protein level.

### 3.2. Causal Effects of Gut Microbiota on MASLD

**Figure 3:**
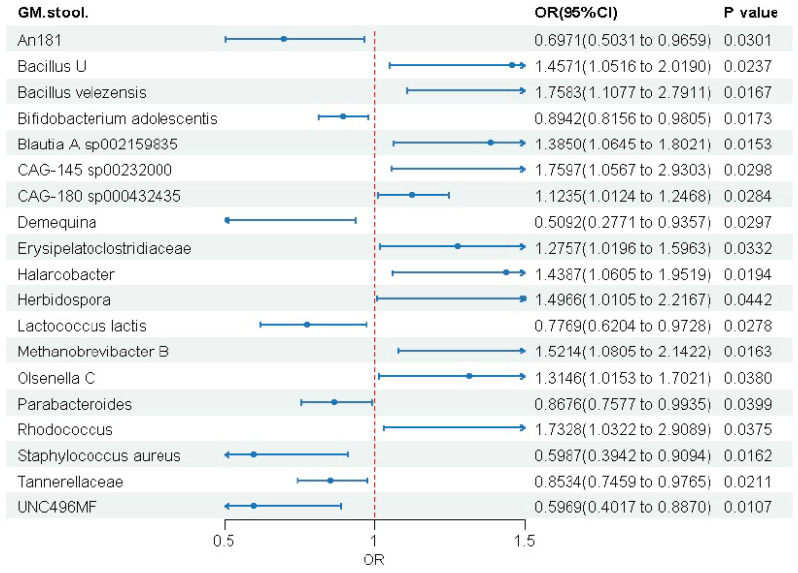
Forest plot showing the Causal associations between gut microbial taxa and MASLD risk in forward MR analysis.

Forward MR analysis identified 19 gut microbial taxa that were significantly associated with MASLD risk out of the 473 taxa tested (Figure 3). The results revealed a clear dichotomous pattern: eight taxa (e.g., *Bifidobacterium adolescentis*, *Lactococcus lactis*) were potential protective microbes (OR < 1), while eleven taxa (e.g., *Bacillus velezensis*, *Blautia A* sp002159835) were potential risk microbes (OR > 1). Detailed results are presented in the forest plot (Figure 3). The F-statistics for all 19 selected gut microbiota were greater than 10, as shown in Supplementary Table S1. Sensitivity analyses confirmed the robustness of our causal inferences (Supplementary Table S4). We found no significant heterogeneity or horizontal pleiotropy (all *p* > 0.05). MR-PRESSO detected no outliers (Supplementary Table S3), and leave-one-out analyses showed stable estimates (Supplementary Figure S6). Results were consistent across MR methods, supporting the primary findings.

**Figure 4:**
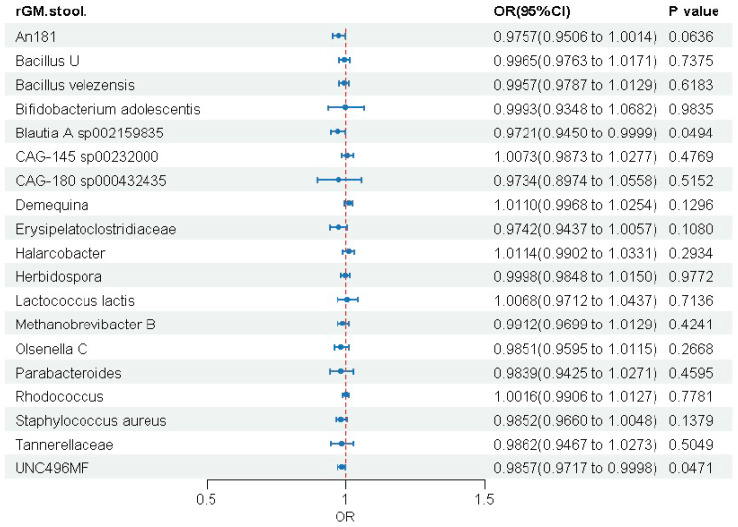
Assessment of reverse causality from MASLD to gut microbiota.

To evaluate reverse causation, we performed reverse MR analyses (Figure 4 and Supplementary Table S5). Results showed that MASLD had no significant causal effect on 17 of the 19 previously identified microbial taxa. Only two taxa (*Blautia A* sp002159835 and UNC496MF) showed significance, but with negligible effect sizes (OR ≈ 1). This finding largely excludes reverse causality. Collectively, our study provides evidence that specific gut microbes have potential causal effects on MASLD risk, and these associations are robust to heterogeneity, pleiotropy, and reverse causation, offering new insights into MASLD pathogenesis.

### 3.3. Causal Effects of m6A Regulatory Genes on Gut Microbiota

We again employed two-sample MR to assess the potential causal relationships between the four identified m6A Regulatory genes (ALKBH3, ALKBH5, CBLL1, and RBM15B) and the 19 gut microbial traits. The IVW method served as the primary approach, with a significance level set at *p* < 0.05.

#### 3.3.1. ALKBH3 Gene

**Figure 5:**
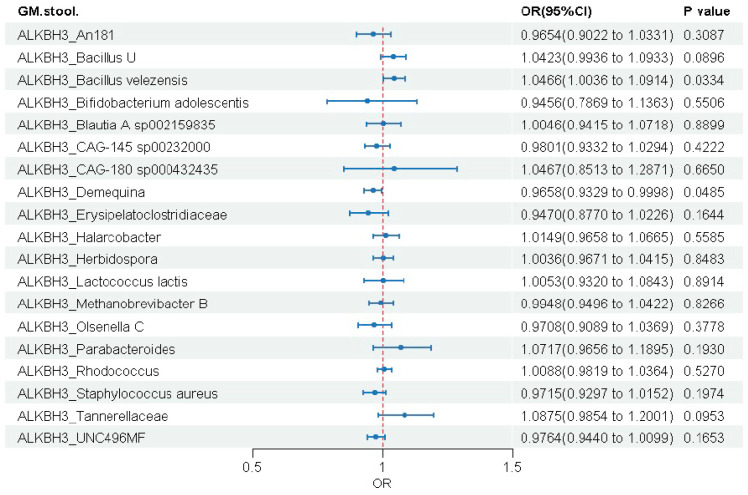
Causal effects of genetically predicted ALKBH3 expression on gut microbial abundance.

MR analysis identified significant causal associations between genetically predicted ALKBH3 expression and two of the 19 gut microbial taxa (Figure 5). Higher ALKBH3 expression was associated with an increase in the abundance of *Bacillus velezensis* (OR = 1.047, 95% CI: 1.004–1.091, *p* = 0.033) and a decrease in the abundance of *Demequina* (OR = 0.966, 95% CI: 0.933–1.000, *p* = 0.049). Sensitivity analyses revealed no significant heterogeneity, horizontal pleiotropy, or outliers (all *p* > 0.05), supporting the robustness of these causal estimates (Supplementary Table S6).

#### 3.3.2. ALKBH5 Gene

**Figure 6:**
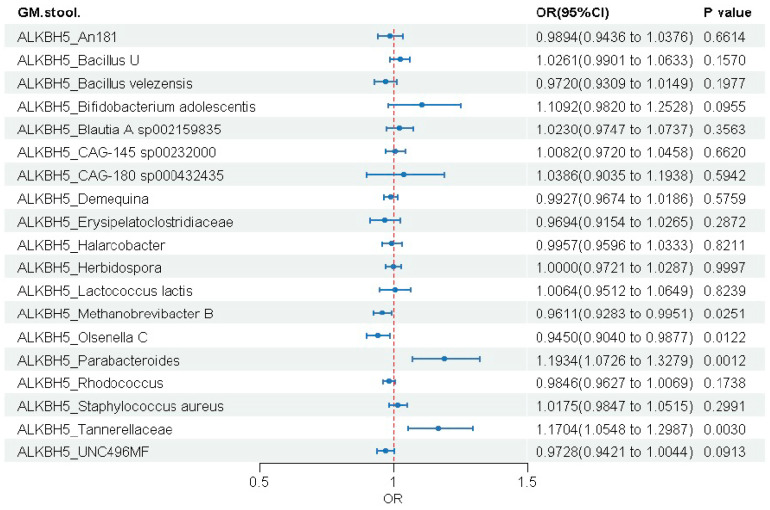
Causal effects of genetically predicted ALKBH5 expression on gut microbial abundance.

ALKBH5 showed the most extensive associations with gut microbiota among all genes analyzed, showing significant causal links with four microbial traits (Figure 6 and Supplementary Table S7). Genetically elevated ALKBH5 expression was associated with reduced abundance of Methanobrevibacter B (OR = 0.961, 95% CI: 0.928–0.995, *p* = 0.025) and Olsenella C (OR = 0.945, 95% CI: 0.904–0.988, *p* = 0.012), but increased abundance of Parabacteroides (OR = 1.193, 95% CI: 1.073–1.328, *p* = 0.001) and Tannerellaceae (OR = 1.170, 95% CI: 1.055–1.299, *p* = 0.003).

#### 3.3.3. CBLL1 Gene

**Figure 7:**
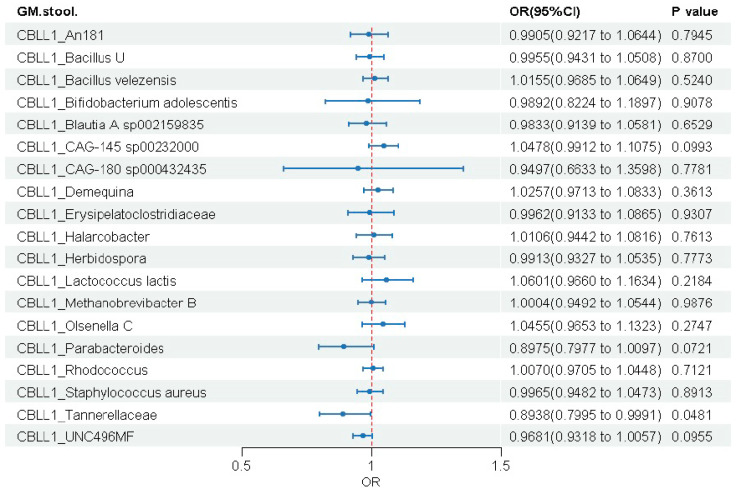
Causal effects of genetically predicted CBLL1 expression on gut microbial abundance.

Among the 19 microbial taxa analyzed, CBLL1 showed a significant causal association with only one taxon (Figure 7 and Supplementary Table S8). Genetically predicted higher CBLL1 expression was associated with a reduced abundance of Tannerellaceae (OR = 0.894, 95% CI: 0.800–1.000, *p* = 0.048).

#### 3.3.4. RBM15B Gene

**Figure 8:**
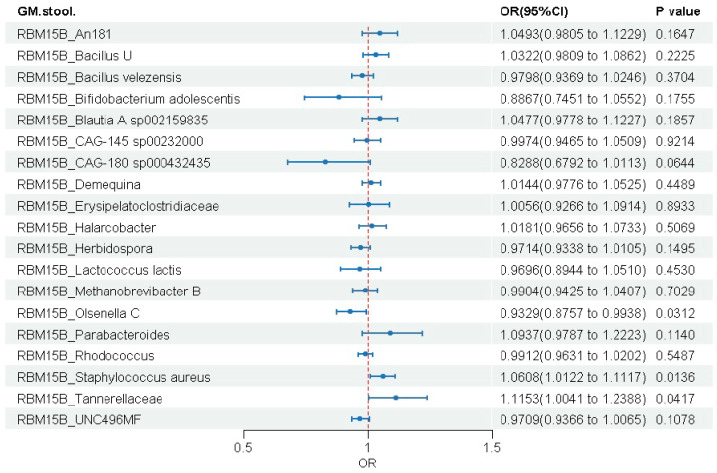
Causal effects of genetically predicted RBM15B expression on gut microbial abundance.

RBM15B expression showed significant associations with three gut microbial taxa (Figure 8 and Supplementary Table S9). Genetically elevated RBM15B expression was associated with reduced abundance of *Olsenella C* (OR = 0.933, 95% CI: 0.876–0.994, *p* = 0.031), but increased abundance of both *Staphylococcus aureus* (OR = 1.061, 95% CI: 1.012–1.112, *p* = 0.014) and Tannerellaceae (OR = 1.115, 95% CI: 1.004–1.239, *p* = 0.042).

#### 3.3.5. Summary of Findings

**Figure 9:**
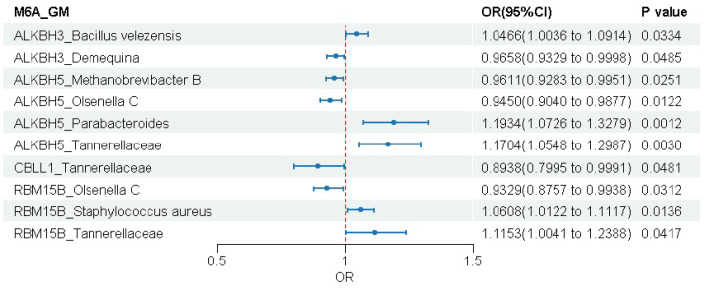
Summary of causal associations between four m6A regulators and seven gut microbial taxa.

In summary, four m6A gene family members (ALKBH3, ALKBH5, CBLL1, RBM15B) demonstrated statistically significant associations with seven gut microbial taxa. To clearly present all significant results, we constructed a forest plot (Figure 9).

### 3.4. Result of Mediation Analysis

**Figure 10:**
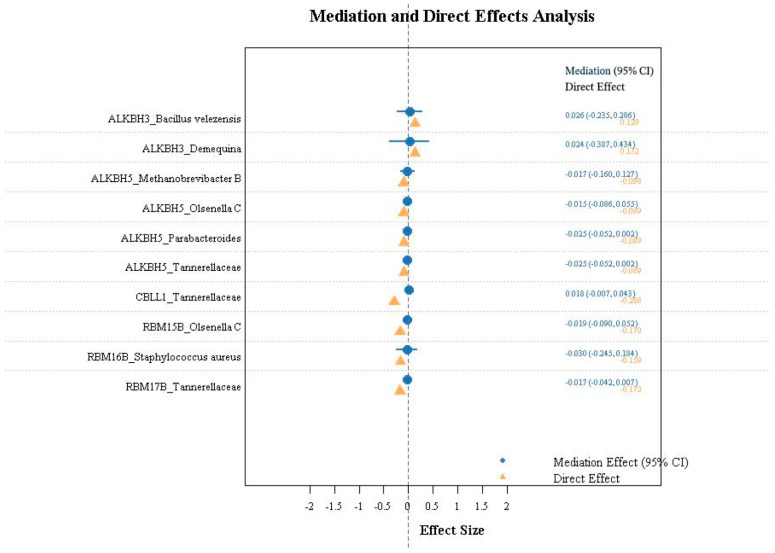
Mediation and direct effects of m6A-microbiota pathways on MASLD risk.

**Figure 11:**
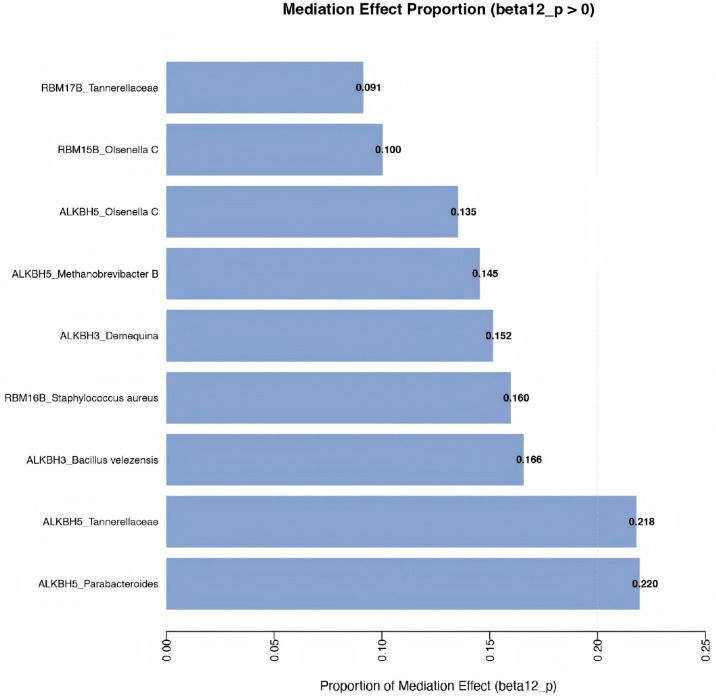
Proportion of the total effect mediated by gut microbiota in m6A-MASLD pathways.

To investigate whether m6A regulators influence MASLD through gut microbiota, we performed mediation analysis. Results showed that among the ten m6A-microbiota combinations examined, most did not show significant indirect mediation effects. Specifically, the indirect effects (β_12_) for all m6A-microbiota pathways were statistically non-significant (all *p* > 0.05), with their 95% confidence intervals including zero. Analysis of the proportion mediated (β_12_/βall) indicated generally low mediation proportions, ranging from 9.14% to 21.9%. The ALKBH5_Parabacteroides and ALKBH5_Tannerellaceae axes showed relatively higher mediation proportions (21.9% and 21.8%), followed by ALKBH3_*Bacillus velezensis* (16.6%) and ALKBH3_*Demequina* (15.2%). The remaining combinations all demonstrated mediation proportions below 16%, with full details provided in Figure 10 and Figure 11. It should be noted that the *p*-values for the ALKBH5 → Parabacteroides and ALKBH5 → Tannerellaceae pathways were 0.068 and 0.069, which did not reach statistical significance at the nominal level. These findings should be interpreted with caution given the absence of significant mediation effects.

## 4. Discussion

Through systematic Mendelian randomization analysis, this study provides the first genetic evidence supporting causal relationships between specific m6A regulators and MASLD. We identified four key genes: ALKBH3 as a risk factor, and ALKBH5, CBLL1, and RBM15B as protective factors. These findings align with emerging evidence that m6A regulators play key roles in lipid metabolism and metabolic diseases [28,29,30]. Notably, the protective role of ALKBH5 is consistent with its reported function in enhancing insulin sensitivity and promoting fatty acid oxidation [31,32]. Conversely, the risk association of ALKBH3—also a demethylase—suggests that m6A demethylases may exert divergent effects depending on tissue context or disease stage.

Interestingly, several well-known m6A regulators previously implicated in lipid metabolism, such as METTL14, METTL5, and FTO, did not show significant causal associations in our MR analysis. This may reflect differences in genetic regulation across tissues (e.g., blood vs. liver) or that their roles in MASLD may be mediated through pathways not captured by cis-eQTLs in blood. The absence of these genes highlights the complexity of m6A-mediated regulation in MASLD pathogenesis and suggests that tissue-specific or post-translational mechanisms may be relevant.

Our mediation analysis, although not reaching strict statistical significance, provides preliminary support for the novel “m6A–gut microbiota–liver” regulatory axis. The ALKBH5 → Parabacteroides → MASLD and ALKBH5 → Tannerellaceae → MASLD pathways showed the highest mediation proportions (21.9% and 21.8%, respectively), suggesting that gut microbiota may partially convey the effects of certain m6A regulators on MASLD risk [31]. This aligns with emerging evidence that altered expression of m6A regulators may directly or indirectly shape gut microbiota composition, thereby contributing to the progression of various diseases [33,34,35]. From a mechanistic perspective, m6A regulators may influence MASLD through multiple interconnected pathways, collectively forming an “m6A-gut microbiota-liver” regulatory network. First, m6A modifications directly regulate the host gene expression profile by altering the stability and translation efficiency of mRNAs involved in intestinal mucosal integrity, immune signaling, and metabolite sensing [36]. Furthermore, metabolites produced by the gut microbiota, such as short-chain fatty acids and bile acids, can feedback to regulate the expression of m6A-related enzymes, establishing a bidirectional regulatory loop between the host and the microbial community [28]. Simultaneously, m6A regulators also modulate cytokine and inflammasome activity, shaping the gut microenvironment and influencing the structure of the microbial community, thereby indirectly regulating intrahepatic lipid metabolism and inflammatory responses [37].

The identification of CBLL1 and RBM15B as protective factors extends beyond their established roles in m6A complex assembly and site recognition, suggesting they may influence hepatic lipid metabolism through RNA stability, splicing, or translation of key metabolic transcripts.

Notably, among all m6A regulators analyzed, CBLL1 exhibited the strongest protective effect against MASLD (OR = 0.763, *p* = 0.001). Interestingly, although both CBLL1 and ALKBH5 were identified as protective factors for MASLD, they showed opposing effects on Tannerellaceae abundance: CBLL1 was associated with reduced Tannerellaceae abundance (OR = 0.894, *p* = 0.048), whereas ALKBH5 was associated with increased abundance of this taxon (OR = 1.170, *p* = 0.003). This discrepancy highlights the functional diversity within the gut microbiota. Tannerellaceae encompasses multiple genera and strains that may exert opposing effects on host metabolism. The current GWAS data are limited to the family-level taxonomic resolution. Moreover, the impact of gut microbiota on disease risk is highly context-dependent, influenced by factors such as diet, host genetics, and microbial community interactions. Therefore, a given microbial taxon may be harmful under certain conditions but benign or even beneficial under others.

Several limitations should be considered. First, blood-derived eQTL data may not fully capture gene regulation in disease-relevant tissues such as the liver or gut. Although blood eQTLs are relevant to MASLD-related systemic immunity and more accessible for clinical translation, future validation using tissue-specific eQTL data is warranted. Second, the lack of species- or strain-level resolution in gut microbiota GWAS data limited functional interpretation. Third, despite an F-statistic >10 for all instruments, the relatively lenient LD clumping threshold (r^2^ < 0.1) and genome-wide significance level (5 × 10^−6^) may have influenced instrument selection. Moreover, the limited number of instruments for certain m6A-related genes (e.g., only seven SNPs for CBLL1) reduced the statistical power of MR-Egger, MR-PRESSO, and mediation analyses, warranting cautious interpretation and further experimental validation. Fourth, the lack of multiple testing correction may inflate false positives. In the analyses of m6A-related genes (24 tests) and gut microbiota (473 tests), we used a nominal threshold of *p* < 0.05, yielding an expected 1.5 and 24.85 false positives. A strict Bonferroni correction (*p* < 1.01 × 10^−4^) would control false positives but may overlook biologically relevant signals due to over-conservatism. Given the exploratory nature of this study, these findings require validation in independent cohorts with rigorous correction or further experimental verification. Finally, the use of European-ancestry datasets may introduce sample overlap and limit generalizability to other populations. Future studies with larger, more diverse cohorts are needed to confirm our findings.

## 5. Conclusions

In summary, this Mendelian randomization study identifies ALKBH3, ALKBH5, CBLL1, and RBM15B as causal factors in MASLD and provides the first genetic evidence supporting a potential mediating role of gut microbiota in the “m6A–gut microbiota–MASLD” axis. Although mediation effects were statistically modest, the pathways such as ALKBH5–Parabacteroides and ALKBH5–Tannerellaceae exhibit notable importance, highlighting an intrinsic connection between epitranscriptomic regulation and microbial ecology in MASLD pathogenesis. These findings offer new insights into the molecular mechanisms underlying MASLD and suggest that targeting specific m6A regulators (e.g., ALKBH5) or their associated gut microbiota (e.g., Parabacteroides) may represent a promising strategy for MASLD, although further functional studies are required.

## Figures and Tables

**Table 1:** Detailed causal estimates of the four m6A regulators significantly associated with MASLD risk.

m6A Regulator	IVs	OR (95% CI)	*p*-Value	Effect Direction
ALKBH3	9	1.168 (1.042–1.309)	0.008	Risk effect
ALKBH5	8	0.892 (0.808–0.984)	0.023	Protective effect
CBLL1	7	0.763 (0.648–0.899)	0.001	Protective effect
RBM15B	8	0.828 (0.728–0.941)	0.004	Protective effect

## Data Availability

The data presented in this study are available in the GWAS Catalog (accession number GCST90091033) at https://www.ebi.ac.uk/gwas/ (accessed on 18 September 2025); the eQTLGen database at https://www.eqtlgen.org/cis-eqtls.html (accessed on 17 September 2025); and the deCODE database at https://www.decode.com/summarydata/ (accessed on 17 September 2025). The gut microbiota GWAS data were derived from the study by Qin et al. [21].

## Supplementary Materials

The following supporting information can be downloaded at: https://www.mdpi.com/article/10.3390/biomedicines14030630/s1. Supplementary Table S1: The R^2^ and F of MR analysis; Supplementary Table S2: 4 alternative MR analysis of Causal Effects of m6A Regulatory Genes on MASLD; Supplementary Table S3: All results of MR analysis (the Heterogeneity *p*-value, Pleiotropy *p*-value, MR-PRESSO *p*-value); Supplementary Table S4: 4 alternative MR analysis of Causal Effects of gut microbiota on MASLD; Supplementary Table S5: 4 alternative MR analysis of reverse MR analyses; Supplementary Table S6: 4 alternative MR analysis of ALKBH3 expression and gut microbiota; Supplementary Table S7: 4 alternative MR analysis of ALKBH5 expression and gut microbiota; Supplementary Table S8: 4 alternative MR analysis of CBLL1 expression and gut microbiota; Supplementary Table S9: 4 alternative MR analysis of RBM15B expression and gut microbiota; Supplementary Figure S1: Identification of genetically regulated m6A regulatory genes; Supplementary Figure S2: MR-Egger analysis of m6A Regulatory Genes on MASLD; Supplementary Figure S3: Leave-one-out analysis of MR analysis of m6A Regulatory Genes on MASLD; Supplementary Figure S4: Funnel plots of MR analysis of m6A Regulatory Genes and MASLD; Supplementary Figure S5: Scatter plots of MR analysis of m6A Regulatory Genes and MASLD; Supplementary Figure S6: Leave-one-out analysis of MR analysis of gut microbiota on MASLD.

## Abbreviations

The following abbreviations are used in this manuscript:

MASLD	Metabolic dysfunction-associated steatotic liver disease
MR	Mendelian randomization
GWAS	genome-wide association studies
MASH	metabolic dysfunction-associated steatohepatitis
m6A	N^6^-methyladenosine
LPS	lipopolysaccharides
SCFAs	short-chain fatty acids
eQTL	expression quantitative trait loci
pQTL	protein quantitative trait loci
NAFLD	Nonalcoholic Fatty Liver Disease
SNPs	single nucleotide polymorphisms
IVs	Instrumental variables
CIs	confidence intervals
ORs	odds ratios
IVW	inverse-variance weighted
MR-PRESSO	MR-Pleiotropy Residual Sum and Outlier

## References

[1] Wong V.W.-S., Ekstedt M., Wong G.L.-H., Hagström H. (2023). Changing epidemiology, global trends and implications for outcomes of NAFLD. J. Hepatol..

[2] Chan W.-K., Chuah K.-H., Rajaram R.B., Lim L.-L., Ratnasingam J., Vethakkan S.R. (2023). Metabolic Dysfunction-Associated Steatotic Liver Disease (MASLD): A State-of-the-Art Review. J. Obes. Metab. Syndr..

[3] Li Y., Yang P., Ye J., Xu Q., Wu J., Wang Y. (2024). Updated mechanisms of MASLD pathogenesis. Lipids Health Dis..

[4] Petri B.J., Klinge C.M. (2023). m6A readers, writers, erasers, and the m6A epitranscriptome in breast cancer. J. Mol. Endocrinol..

[5] Liu X.-M., Zhou J. (2021). Multifaceted regulation of translation by the epitranscriptomic modification N6-methyladenosine. Crit. Rev. Biochem. Mol. Biol..

[6] Boulias K., Greer E.L. (2023). Biological roles of adenine methylation in RNA. Nat. Rev. Genet..

[7] Huang W., Kong D. (2021). The intestinal microbiota as a therapeutic target in the treatment of NAFLD and ALD. Biomed. Pharmacother..

[8] Li F., Ye J., Shao C., Zhong B. (2021). Compositional alterations of gut microbiota in nonalcoholic fatty liver disease patients: A systematic review and Meta-analysis. Lipids Health Dis..

[9] Pabst O., Hornef M.W., Schaap F.G., Cerovic V., Clavel T., Bruns T. (2023). Gut–liver axis: Barriers and functional circuits. Nat. Rev. Gastroenterol. Hepatol..

[10] Sanderson E. (2021). Multivariable Mendelian Randomization and Mediation. Cold Spring Harb. Perspect. Med..

[11] Wang H., Xu P., Yin K., Wang S. (2025). The role of m6A modification during macrophage metabolic reprogramming in human diseases and animal models. Front. Immunol..

[12] Huang X., Yu Z., Tian J., Chen T., Wei A., Mei C., Chen S., Li Y. (2025). m6A RNA modification pathway: Orchestrating fibrotic mechanisms across multiple organs. Brief. Funct. Genom..

[13] Wang H., Han J., Kong H., Ma C., Zhang X.-a. (2025). The Emerging Role of m6A and Programmed Cell Death in Cardiovascular Diseases. Biomolecules.

[14] Shen R., Jiang Z., Wang H., Zheng Z., Jiang X. (2025). Molecular mechanisms of m6A modifications regulating tumor radioresistance. Mol. Med..

[15] Ding S.-Q., Zhang X.-P., Pei J.-P., Bai X., Ma J.-J., Zhang C.-D., Dai D.-Q. (2023). Role of N6-methyladenosine RNA modification in gastric cancer. Cell Death Discov..

[16] Hu X., Lei X., Guo J., Fu W., Sun W., Lu Q., Su W., Xu Q., Tu K. (2022). The Emerging Role of RNA N6-Methyladenosine Modification in Pancreatic Cancer. Front. Oncol..

[17] Shi B., Liu W.-W., Yang K., Jiang G.-M., Wang H. (2022). The role, mechanism, and application of RNA methyltransferase METTL14 in gastrointestinal cancer. Mol. Cancer.

[18] Xu Q., Ren N., Ren L., Yang Y., Pan J., Shang H. (2024). RNA m6A methylation regulators in liver cancer. Cancer Cell Int..

[19] Võsa U., Claringbould A., Westra H.-J., Bonder M.J., Deelen P., Zeng B., Kirsten H., Saha A., Kreuzhuber R., Yazar S. (2021). Large-scale cis- and trans-eQTL analyses identify thousands of genetic loci and polygenic scores that regulate blood gene expression. Nat. Genet..

[20] Ferkingstad E., Sulem P., Atlason B.A., Sveinbjornsson G., Magnusson M.I., Styrmisdottir E.L., Gunnarsdottir K., Helgason A., Oddsson A., Halldorsson B.V. (2021). Large-scale integration of the plasma proteome with genetics and disease. Nat Genet.

[21] Qin Y., Havulinna A.S., Liu Y., Jousilahti P., Ritchie S.C., Tokolyi A., Sanders J.G., Valsta L., Brożyńska M., Zhu Q. (2022). Combined effects of host genetics and diet on human gut microbiota and incident disease in a single population cohort. Nat. Genet..

[22] Ghodsian N., Abner E., Emdin C.A., Gobeil E., Taba N., Haas M.E., Perrot N., Manikpurage H.D., Gagnon E., Bourgault J. (2021). Electronic health record-based genome-wide meta-analysis provides insights on the genetic architecture of non-alcoholic fatty liver disease. Cell Rep. Med..

[23] Zhang G., Cai Y., Liang J., Zhang J., Jing Z., Lv L., Zhang R., Song J., Dang X., Song Q. (2022). Causal relationships between rheumatism and dyslipidemia: A two-sample Mendelian randomization study. Front. Endocrinol..

[24] Skrivankova V.W., Richmond R.C., Woolf B.A.R., Davies N.M., Swanson S.A., VanderWeele T.J., Timpson N.J., Higgins J.P.T., Dimou N., Langenberg C. (2021). Strengthening the reporting of observational studies in epidemiology using mendelian randomisation (STROBE-MR): Explanation and elaboration. BMJ.

[25] Burgess S., Butterworth A., Thompson S.G. (2013). Mendelian randomization analysis with multiple genetic variants using summarized data. Genet. Epidemiol..

[26] Burgess S., Thompson S.G. (2017). Interpreting findings from Mendelian randomization using the MR-Egger method. Eur. J. Epidemiol..

[27] Hemani G., Zheng J., Elsworth B., Wade K.H., Haberland V., Baird D., Laurin C., Burgess S., Bowden J., Langdon R. (2018). The MR-Base platform supports systematic causal inference across the human phenome. eLife.

[28] Chen H., Ren Y., Yu J., Ren J., Zeng Y., Wu Y., Zhang Q., Xiao X. (2026). Crosstalk between the m6A modification and the gut microbiota in lipid metabolism. Microbiol. Res..

[29] Wang Y., Wang Y., Gu J., Su T., Gu X., Feng Y. (2022). The role of RNA m6A methylation in lipid metabolism. Front Endocrinol.

[30] Ming X., Chen S., Li H., Wang Y., Zhou L., Lv Y. (2024). m6A RNA Methylation and Implications for Hepatic Lipid Metabolism. DNA Cell Biol..

[31] Fang M., Ye L., Zhu Y., Huang L., Xu S. (2025). M6A Demethylase ALKBH5 in Human Diseases: From Structure to Mechanisms. Biomolecules.

[32] Aamodt K., Polay M., Salerno Gonzales V.R., Kulkarni R. (2023). 1567-P: Hepatic ALKBH5 Regulation of Glucose and Lipid Metabolism. Diabetes.

[33] Huang P., Liu M., Zhang J., Zhong X., Zhong C. (2023). YTHDF1 Attenuates TBI-Induced Brain-Gut Axis Dysfunction in Mice. Int. J. Mol. Sci..

[34] Sun L., Ma L., Zhang H., Cao Y., Wang C., Hou N., Huang N., von Deneen K.M., Zhao C., Shi Y. (2019). Fto Deficiency Reduces Anxiety- and Depression-Like Behaviors in Mice via Alterations in Gut Microbiota. Theranostics.

[35] Wang H., Han J., Zhang X.-A. (2025). Interplay of m6A RNA methylation and gut microbiota in modulating gut injury. Gut Microbes.

[36] Chen Y., Lei J., He S. (2021). m(6)A Modification Mediates Mucosal Immune Microenvironment and Therapeutic Response in Inflammatory Bowel Disease. Front. Cell Dev. Biol..

[37] Hu Q., Gao Y., Xie Y., Li D., An T., Chen L., Ji W., Jin Y., Long J., Yang H. (2023). Mechanism of RNA m(6) A methylation modification regulating NLRP3 inflammasome activation for hand, foot, and mouth disease progression. J. Med. Virol..

