# Supplementary material for: Mediation Role of Gut Microbiota in the Causal Relationship Between m6A Regulatory Genes and Metabolic Dysfunction-Associated Steatotic Liver Disease: A Mendelian Randomization Study
Source: Biomedicines. 2026 Mar 11;14(3):630. doi: 10.3390/biomedicines14030630 (PMC13023808; doi:10.3390/biomedicines14030630)

Supplementary Figure S1. Identification of genetically regulated m6A regulatory genes.

A. Using cis-eQTL data from the eQTLGen consortium (N = 15,695), 24 genes were identified with significant cis-eQTLs within ±100 kb of their genomic positions ($p < 5 \times 10^{-8}$). B. Using pQTL data from the deCODE database (N = 1,615), 1 gene (ALKBH3) was found to have a significant pQTL.

Supplementary Figure S2. MR-Egger analysis of m6A Regulatory Genes on MASLD.

Supplementary Figure S3. Leave-one-out analysis of MR analysis of m6A Regulatory Genes on MASLD.

Supplementary Figure S4. Funnel plots of MR analysis of m6A Regulatory Genes and MASLD.

Supplementary Figure S5: Scatter plots of MR analysis of m6A Regulatory Genes and MASLD.

Supplementary Figure S6: Leave-one-out analysis of MR analysis of gut microbiota on MASLD.

**Supplementary Figure S1. Identification of genetically regulated m6A regulatory genes.**

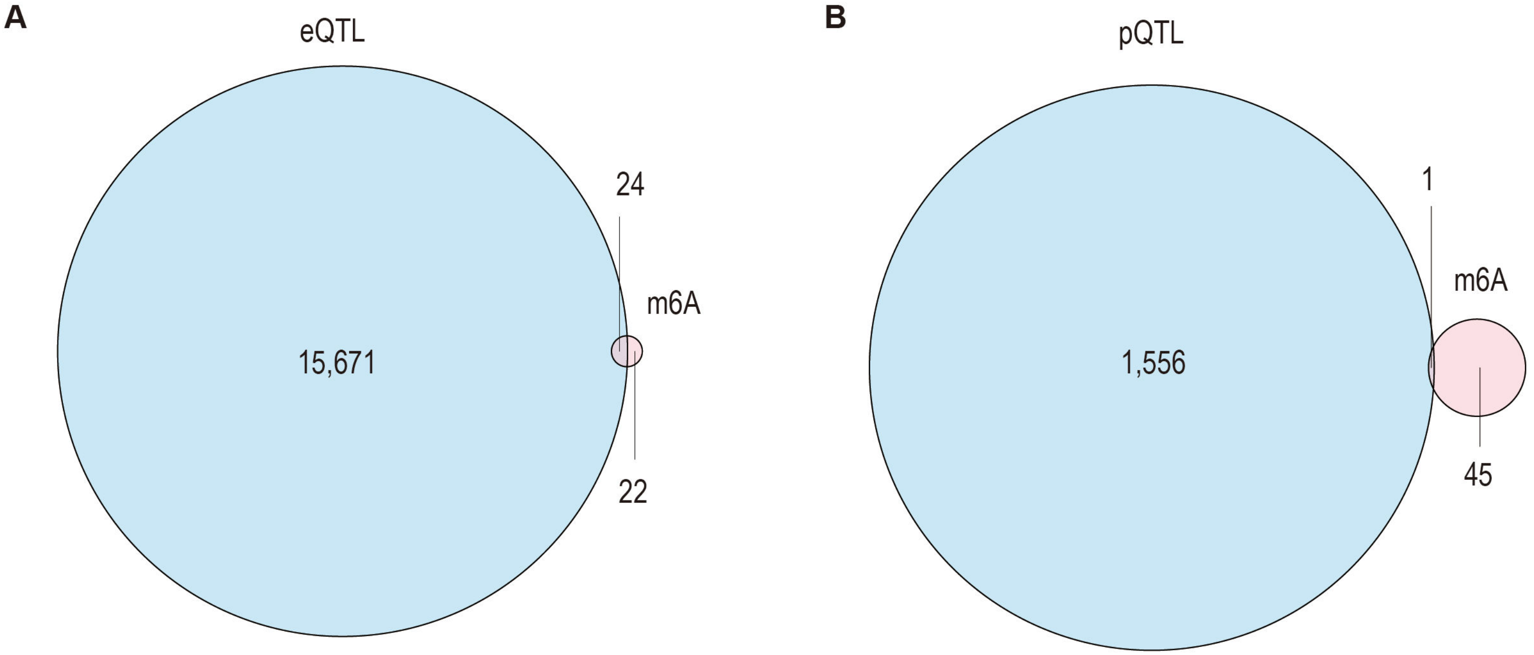

# Supplementary Figure S2. MR-Egger analysis of m6A Regulatory Genes on MASLD.

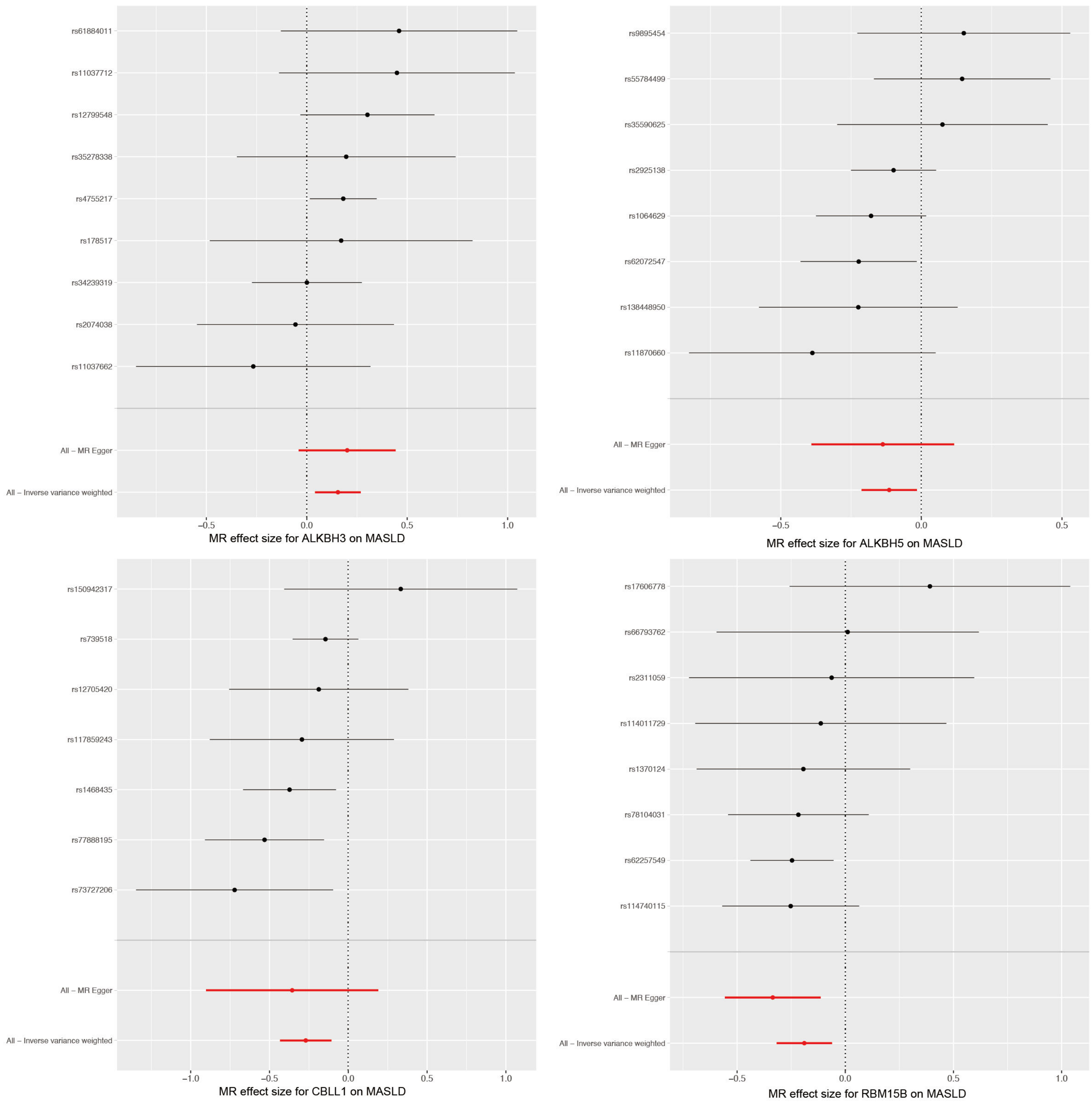

# Supplementary Figure S3. Leave-one-out analysis of MR analysis of m6A Regulatory Genes on MASLD.

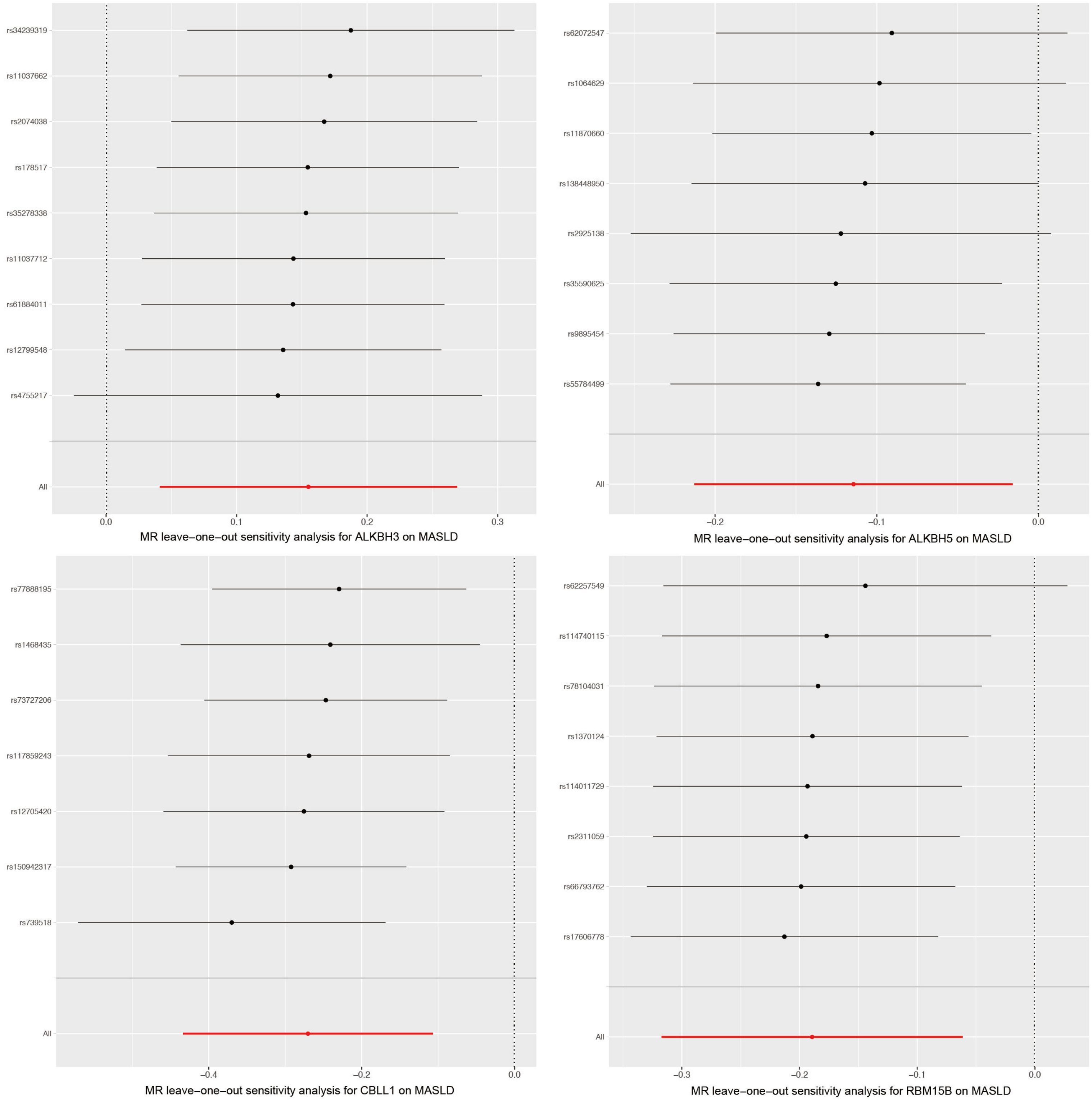

# Supplementary Figure S4. Funnel plots of MR analysis of m6A Regulatory Genes and MASLD.

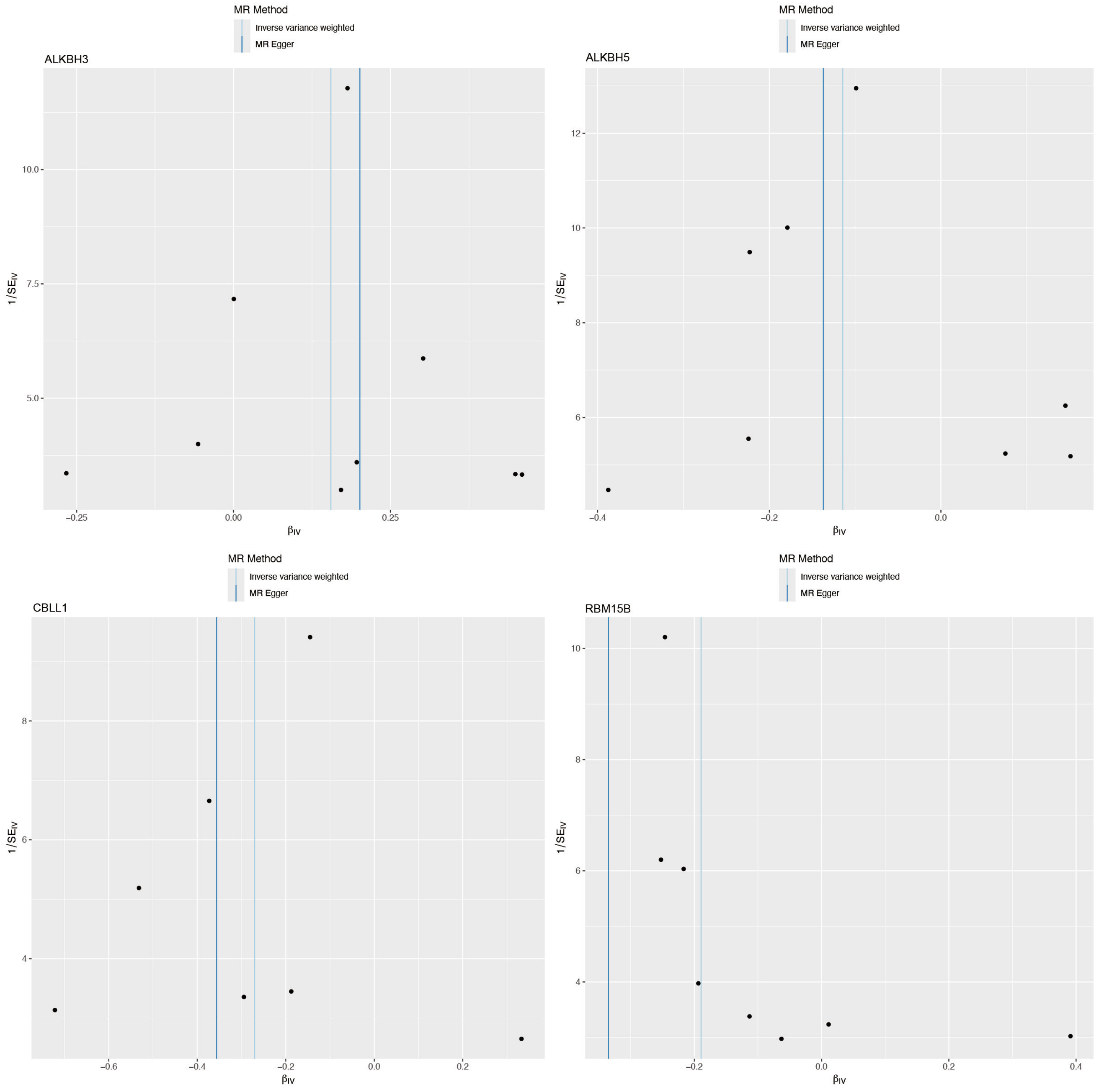

# Supplementary Figure S5: Scatter plots of MR analysis of m6A Regulatory Genes and MASLD.

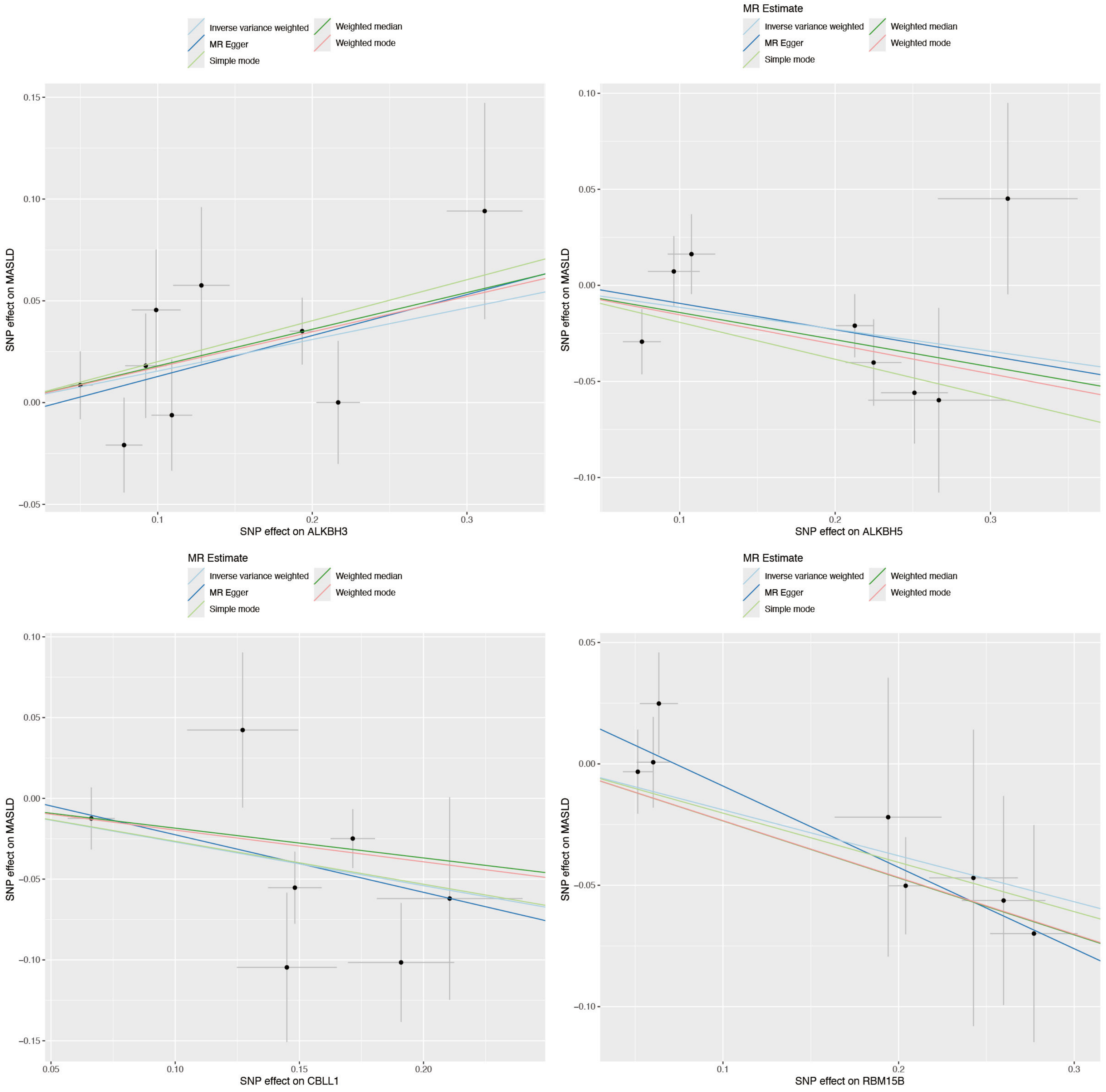

**Supplementary Figure S6: Leave-one-out analysis of MR analysis of gut microbiota on MASLD.**

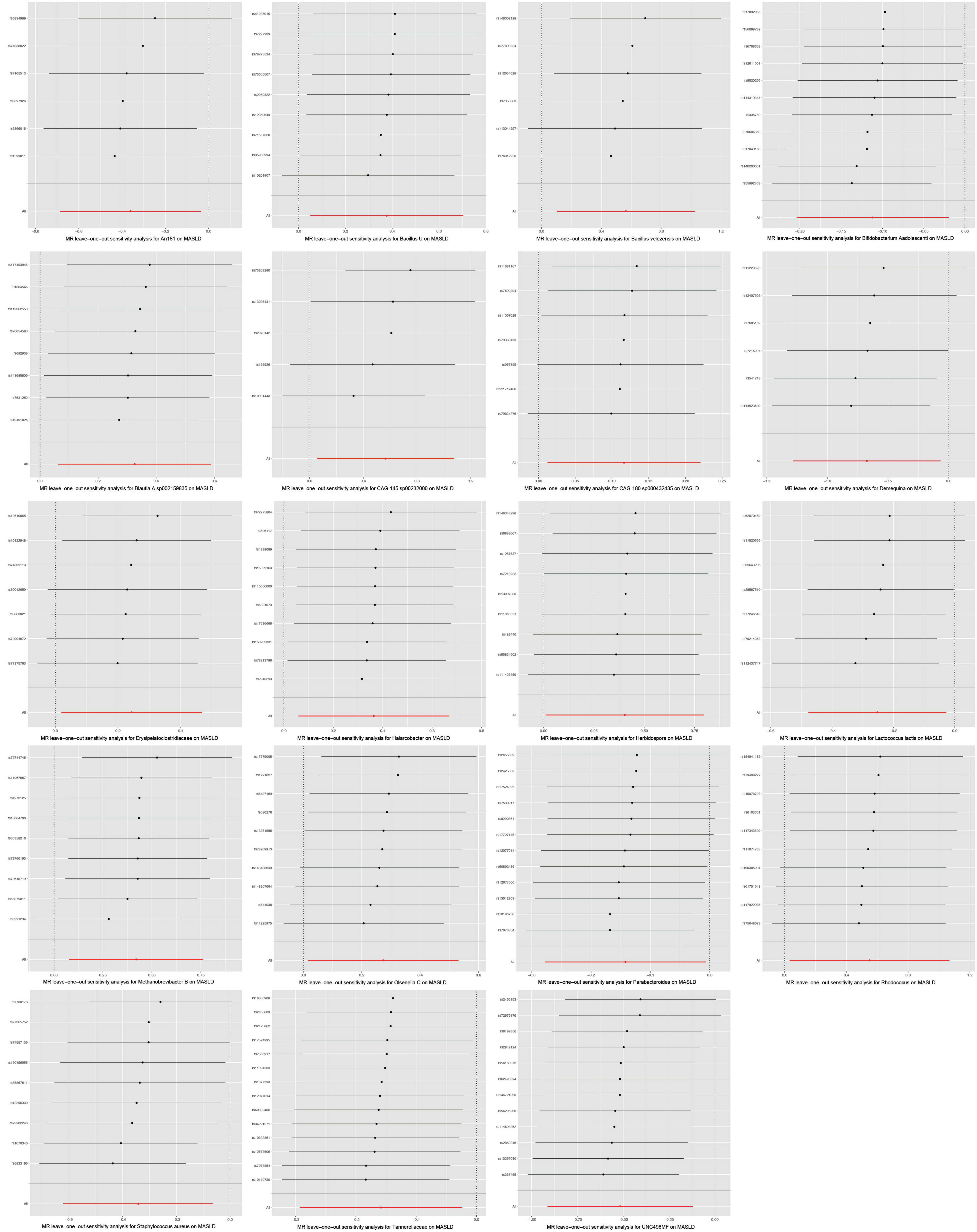

Supplement: Supplementary file 1 [file biomedicines-14-00630-s001.zip › Supplementary Figure.pdf]
