# Supplementary material for: Mediation Role of Gut Microbiota in the Causal Relationship Between m6A Regulatory Genes and Metabolic Dysfunction-Associated Steatotic Liver Disease: A Mendelian Randomization Study
Source: Biomedicines. 2026 Mar 11;14(3):630. doi: 10.3390/biomedicines14030630 (PMC13023808; doi:10.3390/biomedicines14030630)
Supplement: Supplementary file 1 [file biomedicines-14-00630-s001.zip › Supplementary Table S1_The R2 and F of MR analysis.pdf]

Supplementary Table s1.1 The R^2, F and meanf of m6A.exposure_MASLD.outcome.

m6A.exposure_MASLD.outcome	SNP	R^2	F	meanf
ALKBH1_MASLD	rs117620483	0.001	34.479	126.143
ALKBH1_MASLD	rs12878486	0.003	91.483	126.143
ALKBH1_MASLD	rs140089337	0.002	38.393	126.143
ALKBH1_MASLD	rs146875663	0.016	51.56	126.143
ALKBH1_MASLD	rs17751746	0.001	34.794	126.143
ALKBH1_MASLD	rs2006235	0.002	63.46	126.143
ALKBH1_MASLD	rs4903649	0.002	45.361	126.143
ALKBH1_MASLD	rs55692483	0.008	241.149	126.143
ALKBH1_MASLD	rs56412551	0.022	705.267	126.143
ALKBH1_MASLD	rs731346	0.001	30.545	126.143
ALKBH1_MASLD	rs74560170	0.001	34.024	126.143
ALKBH1_MASLD	rs74760472	0.004	133.228	126.143
ALKBH1_MASLD	rs76023729	0.006	186.062	126.143
ALKBH1_MASLD	rs8022045	0.003	76.204	126.143
ALKBH3_MASLD	rs11037662	0.001	43.47	140.538
ALKBH3_MASLD	rs11037712	0.002	49.297	140.538
ALKBH3_MASLD	rs12799548	0.005	162.139	140.538
ALKBH3_MASLD	rs178517	0.001	37.882	140.538
ALKBH3_MASLD	rs2074038	0.002	69.148	140.538
ALKBH3_MASLD	rs34239319	0.008	237.265	140.538
ALKBH3_MASLD	rs35278338	0.002	47.917	140.538
ALKBH3_MASLD	rs4755217	0.018	578.573	140.538
ALKBH3_MASLD	rs61884011	0.001	39.153	140.538
ALKBH5_MASLD	rs1064629	0.011	157.202	100.521
ALKBH5_MASLD	rs11870660	0.003	38.279	100.521
ALKBH5_MASLD	rs138448950	0.003	34.46	100.521
ALKBH5_MASLD	rs2925138	0.021	305.952	100.521
ALKBH5_MASLD	rs35590625	0.004	33.503	100.521
ALKBH5_MASLD	rs55784499	0.004	47.904	100.521
ALKBH5_MASLD	rs62072547	0.012	136.964	100.521
ALKBH5_MASLD	rs9895454	0.003	49.902	100.521
CBLL1_MASLD	rs117859243	0.002	51.53	117.243
CBLL1_MASLD	rs12705420	0.002	48.839	117.243
CBLL1_MASLD	rs1468435	0.006	188.045	117.243
CBLL1_MASLD	rs150942317	0.001	32.38	117.243
CBLL1_MASLD	rs73727206	0.002	52.066	117.243
CBLL1_MASLD	rs739518	0.012	368.068	117.243
CBLL1_MASLD	rs77888195	0.003	79.773	117.243
CEBPZ_MASLD	rs10188177	0.044	1447.116	150.649
CEBPZ_MASLD	rs1024816	0.001	39.544	150.649
CEBPZ_MASLD	rs10724	0.004	109.26	150.649
CEBPZ_MASLD	rs112168902	0.001	41.818	150.649
CEBPZ_MASLD	rs115907036	0.002	54.067	150.649
CEBPZ_MASLD	rs12712516	0.001	40.877	150.649
CEBPZ_MASLD	rs13423928	0.011	336.463	150.649
CEBPZ_MASLD	rs138255475	0.002	43.401	150.649
CEBPZ_MASLD	rs143354126	0.005	163.487	150.649
CEBPZ_MASLD	rs143503814	0.001	33.609	150.649
CEBPZ_MASLD	rs147408352	0.005	120.551	150.649
CEBPZ_MASLD	rs149321780	0.002	36.008	150.649
CEBPZ_MASLD	rs17038876	0.004	115.484	150.649
CEBPZ_MASLD	rs187485016	0.001	35.222	150.649
CEBPZ_MASLD	rs2302653	0.003	98.156	150.649
CEBPZ_MASLD	rs2372708	0.001	33.554	150.649
CEBPZ_MASLD	rs2373000	0.014	458.907	150.649
CEBPZ_MASLD	rs35118709	0.004	111.436	150.649
CEBPZ_MASLD	rs35448037	0.006	142.762	150.649
CEBPZ_MASLD	rs4648178	0.003	81.097	150.649
CEBPZ_MASLD	rs4670618	0.003	88.878	150.649
CEBPZ_MASLD	rs55823009	0.002	56.756	150.649
CEBPZ_MASLD	rs56868575	0.002	47.03	150.649
CEBPZ_MASLD	rs6709930	0.01	325.774	150.649
CEBPZ_MASLD	rs6748659	0.002	50.459	150.649
CEBPZ_MASLD	rs72792854	0.004	117.463	150.649
CEBPZ_MASLD	rs72875755	0.003	102.25	150.649
CEBPZ_MASLD	rs74417714	0.002	67.376	150.649
CEBPZ_MASLD	rs7561725	0.001	32.488	150.649
CEBPZ_MASLD	rs7568641	0.001	41.407	150.649
CEBPZ_MASLD	rs77321436	0.009	255.911	150.649
CEBPZ_MASLD	rs77741235	0.006	162.019	150.649
CEBPZ_MASLD	rs78304212	0.006	171.795	150.649
CEBPZ_MASLD	rs79889719	0.002	59.636	150.649
FTO_MASLD	rs10521305	0.007	195.817	100.275
FTO_MASLD	rs10852525	0.001	31.541	100.275
FTO_MASLD	rs112093224	0.002	65.05	100.275
FTO_MASLD	rs1344498	0.002	49.38	100.275
FTO_MASLD	rs1344501	0.015	467.23	100.275
FTO_MASLD	rs17818800	0.002	62.197	100.275
FTO_MASLD	rs35013387	0.001	30.596	100.275
FTO_MASLD	rs35089292	0.001	32.863	100.275
FTO_MASLD	rs73617811	0.002	69.793	100.275
FTO_MASLD	rs8061064	0.002	72.61	100.275
FTO_MASLD	rs9934773	0.002	72.436	100.275
FTO_MASLD	rs9972717	0.002	53.783	100.275
KIAA1429_MASLD	rs10104041	0.008	262.942	76.146
KIAA1429_MASLD	rs17716313	0.002	72.443	76.146
KIAA1429_MASLD	rs17716607	0.001	30.406	76.146
KIAA1429_MASLD	rs4735305	0.001	43.379	76.146
KIAA1429_MASLD	rs72676941	0.001	29.747	76.146
KIAA1429_MASLD	rs7386115	0.001	35.695	76.146

m6A.exposure_MASLD.outcome	SNP	R^2	F	meanf
KIAA1429_MASLD	rs7820441	0.002	58.409	76.146
METTL5_MASLD	rs113883537	0.002	52.947	185.299
METTL5_MASLD	rs12464221	0.001	31.648	185.299
METTL5_MASLD	rs13027800	0.003	81.22	185.299
METTL5_MASLD	rs2161916	0.001	31.252	185.299
METTL5_MASLD	rs35417312	0.027	862.078	185.299
METTL5_MASLD	rs56154841	0.005	161.788	185.299
METTL5_MASLD	rs9653237	0.002	76.159	185.299
METTL7A_MASLD	rs11169621	0.007	221.867	194.65
METTL7A_MASLD	rs117407215	0.003	72.898	194.65
METTL7A_MASLD	rs12815367	0.002	69.225	194.65
METTL7A_MASLD	rs12819920	0.035	1149.036	194.65
METTL7A_MASLD	rs141423427	0.02	522.964	194.65
METTL7A_MASLD	rs146769945	0.002	45.316	194.65
METTL7A_MASLD	rs149372476	0.004	40.247	194.65
METTL7A_MASLD	rs151297538	0.002	39.022	194.65
METTL7A_MASLD	rs3190077	0.008	257.095	194.65
METTL7A_MASLD	rs3742065	0.002	63.236	194.65
METTL7A_MASLD	rs55962545	0.001	33.745	194.65
METTL7A_MASLD	rs61932889	0.005	157.432	194.65
METTL7A_MASLD	rs6580812	0.003	94.519	194.65
METTL7A_MASLD	rs73088639	0.001	29.867	194.65
METTL7A_MASLD	rs73096828	0.011	340.012	194.65
METTL7A_MASLD	rs75680554	0.001	33.105	194.65
METTL7A_MASLD	rs76637716	0.003	89.326	194.65
METTL7A_MASLD	rs78362716	0.008	244.785	194.65
METTL14_MASLD	rs10021843	0.002	58.9	81.92
METTL14_MASLD	rs113437485	0.002	71.264	81.92
METTL14_MASLD	rs114856557	0.001	29.838	81.92
METTL14_MASLD	rs17865120	0.001	31.497	81.92
METTL14_MASLD	rs1963384	0.002	49.975	81.92
METTL14_MASLD	rs4428355	0.002	50.346	81.92
METTL14_MASLD	rs62328063	0.001	31.952	81.92
METTL14_MASLD	rs6828070	0.009	294.739	81.92
METTL14_MASLD	rs6849210	0.002	62.836	81.92
METTL14_MASLD	rs73842212	0.006	188.02	81.92
METTL14_MASLD	rs960737	0.001	31.759	81.92
RBM15_MASLD	rs116568171	0.004	41.896	127.48
RBM15_MASLD	rs12097823	0.024	347.714	127.48
RBM15_MASLD	rs185790525	0.002	32.938	127.48
RBM15_MASLD	rs3768460	0.013	183.452	127.48
RBM15_MASLD	rs77102212	0.002	31.399	127.48
RBM15B_MASLD	rs114011729	0.002	40.717	116.63
RBM15B_MASLD	rs114740115	0.005	123.889	116.63
RBM15B_MASLD	rs1370124	0.003	92.448	116.63
RBM15B_MASLD	rs17606778	0.001	34.239	116.63
RBM15B_MASLD	rs2311059	0.001	36.849	116.63
RBM15B_MASLD	rs62257549	0.014	443.722	116.63
RBM15B_MASLD	rs66793762	0.001	41.31	116.63
RBM15B_MASLD	rs78104031	0.004	119.869	116.63
SMAD3_MASLD	rs10163040	0.01	290.082	108.051
SMAD3_MASLD	rs11629874	0.001	45.473	108.051
SMAD3_MASLD	rs11635753	0.004	141.339	108.051
SMAD3_MASLD	rs117966956	0.001	34.372	108.051
SMAD3_MASLD	rs17294280	0.002	60.557	108.051
SMAD3_MASLD	rs1992217	0.005	172.809	108.051
SMAD3_MASLD	rs34944378	0.001	31.79	108.051
SMAD3_MASLD	rs7169630	0.003	87.981	108.051
WTAP_MASLD	rs12190141	0.037	1071.975	227.808
WTAP_MASLD	rs12193769	0.01	234.958	227.808
WTAP_MASLD	rs56130537	0.001	39.528	227.808
WTAP_MASLD	rs56762310	0.002	56.646	227.808
WTAP_MASLD	rs7755435	0.001	36.103	227.808
WTAP_MASLD	rs8191745	0.001	37.434	227.808
WTAP_MASLD	rs876381	0.003	93.3	227.808
WTAP_MASLD	rs9346795	0.008	252.524	227.808
YTHDF3_MASLD	rs10107109	0.004	99.593	150.259
YTHDF3_MASLD	rs16929963	0.004	114.039	150.259
YTHDF3_MASLD	rs56347263	0.004	106.29	150.259
YTHDF3_MASLD	rs62510126	0.002	54.812	150.259
YTHDF3_MASLD	rs6993229	0.021	569.596	150.259
YTHDF3_MASLD	rs7460072	0.003	72.593	150.259
YTHDF3_MASLD	rs79701164	0.001	34.889	150.259

Supplementary Table s1.2 The R2, F and meanf of GM.exposure_MASLD.outcome

GM.exposure_MASLD.outcome	SNP	R2	F	meanf
An181_MASLD	rs1599011	0.004	21.29	23.437
An181_MASLD	rs6697926	0.005	29.757	23.437
An181_MASLD	rs6834989	0.004	21.308	23.437
An181_MASLD	rs6866016	0.004	24.251	23.437
An181_MASLD	rs7165513	0.004	22.458	23.437
An181_MASLD	rs74838822	0.004	21.556	23.437
Bacillus U_MASLD	rs10201907	0.006	33.018	25.477
Bacillus U_MASLD	rs12350649	0.004	22.695	25.477
Bacillus U_MASLD	rs2269322	0.005	27.647	25.477
Bacillus U_MASLD	rs35908994	0.004	22.795	25.477
Bacillus U_MASLD	rs41285010	0.004	24.214	25.477
Bacillus U_MASLD	rs71597328	0.005	29.157	25.477
Bacillus U_MASLD	rs7297939	0.004	24.286	25.477
Bacillus U_MASLD	rs73655567	0.004	22.244	25.477
Bacillus U_MASLD	rs78775534	0.004	23.234	25.477
Bacillus velezensis_MASLD	rs113044297	0.005	30.536	24.435
Bacillus velezensis_MASLD	rs12634628	0.004	22.718	24.435
Bacillus velezensis_MASLD	rs146002128	0.004	21.223	24.435
Bacillus velezensis_MASLD	rs7358063	0.004	21.049	24.435
Bacillus velezensis_MASLD	rs76812958	0.005	30.008	24.435
Bacillus velezensis_MASLD	rs77899924	0.004	21.078	24.435
Bifidobacterium adolescentis_MASLD	rs114319347	0.004	22.327	22.58
Bifidobacterium adolescentis_MASLD	rs12611001	0.004	23.292	22.58
Bifidobacterium adolescentis_MASLD	rs140200601	0.004	21.768	22.58
Bifidobacterium adolescentis_MASLD	rs17092955	0.004	23.659	22.58
Bifidobacterium adolescentis_MASLD	rs17849163	0.004	22.823	22.58
Bifidobacterium adolescentis_MASLD	rs335752	0.004	21.745	22.58
Bifidobacterium adolescentis_MASLD	rs55892303	0.004	22.813	22.58
Bifidobacterium adolescentis_MASLD	rs56098739	0.004	25.319	22.58
Bifidobacterium adolescentis_MASLD	rs6768653	0.004	21.044	22.58
Bifidobacterium adolescentis_MASLD	rs78686363	0.004	21.211	22.58
Bifidobacterium adolescentis_MASLD	rs9520255	0.004	22.38	22.58
Blautia A sp002159835_MASLD	rs10491026	0.004	21.399	23.952
Blautia A sp002159835_MASLD	rs112362553	0.004	21.146	23.952
Blautia A sp002159835_MASLD	rs117493946	0.005	27.498	23.952
Blautia A sp002159835_MASLD	rs1364546	0.004	22.713	23.952
Blautia A sp002159835_MASLD	rs141685809	0.004	22.895	23.952
Blautia A sp002159835_MASLD	rs692938	0.005	29.834	23.952
Blautia A sp002159835_MASLD	rs76054583	0.004	20.938	23.952
Blautia A sp002159835_MASLD	rs7631252	0.004	25.192	23.952
CAG-145 sp00232000_MASLD	rs10931443	0.006	38.969	24.85
CAG-145 sp00232000_MASLD	rs13025431	0.004	21.374	24.85
CAG-145 sp00232000_MASLD	rs156926	0.004	21.594	24.85
CAG-145 sp00232000_MASLD	rs2073142	0.004	21.099	24.85
CAG-145 sp00232000_MASLD	rs73203290	0.004	21.213	24.85
CAG-180 sp000432435_MASLD	rs11057029	0.004	25.665	23.305
CAG-180 sp000432435_MASLD	rs111717438	0.004	21.278	23.305
CAG-180 sp000432435_MASLD	rs11681167	0.004	26.694	23.305
CAG-180 sp000432435_MASLD	rs7599804	0.004	23.39	23.305
CAG-180 sp000432435_MASLD	rs79338453	0.004	22.193	23.305
CAG-180 sp000432435_MASLD	rs79834276	0.004	22.636	23.305
CAG-180 sp000432435_MASLD	rs967895	0.004	21.28	23.305
Demequina_MASLD	rs11223695	0.004	24.001	24.952
Demequina_MASLD	rs114523689	0.005	30.147	24.952
Demequina_MASLD	rs12407592	0.004	26.711	24.952
Demequina_MASLD	rs541713	0.004	25.638	24.952
Demequina_MASLD	rs7216027	0.004	21.139	24.952
Demequina_MASLD	rs7605169	0.004	22.077	24.952
Erysipelatoclostridiaceae_MASLD	rs10122948	0.004	21.057	22.822
Erysipelatoclostridiaceae_MASLD	rs12510065	0.004	22.45	22.822
Erysipelatoclostridiaceae_MASLD	rs3863021	0.004	22.494	22.822
Erysipelatoclostridiaceae_MASLD	rs71375763	0.005	30.839	22.822
Erysipelatoclostridiaceae_MASLD	rs72904672	0.003	20.905	22.822
Erysipelatoclostridiaceae_MASLD	rs74365113	0.003	20.864	22.822
Erysipelatoclostridiaceae_MASLD	rs80042659	0.004	21.142	22.822
Halarcobacter_MASLD	rs115056000	0.004	21.585	23.542
Halarcobacter_MASLD	rs150202331	0.005	31.486	23.542
Halarcobacter_MASLD	rs16899163	0.004	25.682	23.542
Halarcobacter_MASLD	rs17536666	0.004	23.205	23.542
Halarcobacter_MASLD	rs2243293	0.004	21.163	23.542
Halarcobacter_MASLD	rs4399689	0.004	22.46	23.542
Halarcobacter_MASLD	rs596117	0.004	23.037	23.542
Halarcobacter_MASLD	rs6831073	0.004	21.173	23.542
Halarcobacter_MASLD	rs72775804	0.004	24.437	23.542
Halarcobacter_MASLD	rs78213796	0.004	21.197	23.542
Herbidospora_MASLD	rs111423259	0.004	21.687	23.195
Herbidospora_MASLD	rs11965551	0.003	20.87	23.195
Herbidospora_MASLD	rs1257637	0.004	22.502	23.195
Herbidospora_MASLD	rs13097088	0.005	29.812	23.195
Herbidospora_MASLD	rs146343298	0.004	22.413	23.195
Herbidospora_MASLD	rs482446	0.005	27.779	23.195
Herbidospora_MASLD	rs55834502	0.004	21.574	23.195
Herbidospora_MASLD	rs6888067	0.003	20.85	23.195
Herbidospora_MASLD	rs7210922	0.004	21.271	23.195
Lactococcus lactis_MASLD	rs112437747	0.005	31.194	23.889
Lactococcus lactis_MASLD	rs11520695	0.005	27.408	23.889
Lactococcus lactis_MASLD	rs28567510	0.004	23.417	23.889
Lactococcus lactis_MASLD	rs35643205	0.004	20.99	23.889
Lactococcus lactis_MASLD	rs62070469	0.004	21.383	23.889
Lactococcus lactis_MASLD	rs77246646	0.004	21.708	23.889

GM.exposure_MASLD.outcome	SNP	R2	F	meanf
Lactococcus lactis_MASLD	rs79214353	0.004	21.123	23.889
Methanobrevibacter B_MASLD	rs11067667	0.004	21.586	23.675
Methanobrevibacter B_MASLD	rs13064706	0.004	23.435	23.675
Methanobrevibacter B_MASLD	rs35358216	0.004	21.352	23.675
Methanobrevibacter B_MASLD	rs4973125	0.004	24.341	23.675
Methanobrevibacter B_MASLD	rs55879811	0.004	22.478	23.675
Methanobrevibacter B_MASLD	rs72648719	0.005	29.982	23.675
Methanobrevibacter B_MASLD	rs72744746	0.004	22.883	23.675
Methanobrevibacter B_MASLD	rs72766193	0.004	22.819	23.675
Methanobrevibacter B_MASLD	rs9991294	0.004	24.196	23.675
Olsenella C_MASLD	rs11225975	0.004	22.363	23.835
Olsenella C_MASLD	rs13251886	0.004	21.965	23.835
Olsenella C_MASLD	rs143498649	0.004	22.089	23.835
Olsenella C_MASLD	rs148907664	0.004	21.016	23.835
Olsenella C_MASLD	rs17375005	0.004	25.045	23.835
Olsenella C_MASLD	rs1991027	0.004	21.879	23.835
Olsenella C_MASLD	rs544038	0.006	36.777	23.835
Olsenella C_MASLD	rs6497169	0.004	22.507	23.835
Olsenella C_MASLD	rs680276	0.004	23.165	23.835
Olsenella C_MASLD	rs78269913	0.004	21.544	23.835
Parabacteroides_MASLD	rs10180730	0.003	20.875	23.293
Parabacteroides_MASLD	rs12077014	0.004	21.317	23.293
Parabacteroides_MASLD	rs12673506	0.006	33.274	23.293
Parabacteroides_MASLD	rs13012303	0.003	20.894	23.293
Parabacteroides_MASLD	rs17524995	0.004	23.652	23.293
Parabacteroides_MASLD	rs17727145	0.004	21.052	23.293
Parabacteroides_MASLD	rs2425862	0.004	20.966	23.293
Parabacteroides_MASLD	rs2655609	0.004	22.774	23.293
Parabacteroides_MASLD	rs2791577	0.004	21.784	23.293
Parabacteroides_MASLD	rs4843380	0.004	21.676	23.293
Parabacteroides_MASLD	rs60892486	0.004	22.551	23.293
Parabacteroides_MASLD	rs7073854	0.004	22.293	23.293
Parabacteroides_MASLD	rs7580217	0.005	30.685	23.293
Parabacteroides_MASLD	rs9290864	0.004	22.312	23.293
Rhodococcus_MASLD	rs10078793	0.005	31.126	23.514
Rhodococcus_MASLD	rs11074733	0.004	23.402	23.514
Rhodococcus_MASLD	rs117343599	0.004	22.411	23.514
Rhodococcus_MASLD	rs117922985	0.004	22.113	23.514
Rhodococcus_MASLD	rs184941182	0.004	24.616	23.514
Rhodococcus_MASLD	rs190385094	0.004	20.993	23.514
Rhodococcus_MASLD	rs6103661	0.004	21.386	23.514
Rhodococcus_MASLD	rs61751543	0.004	24.037	23.514
Rhodococcus_MASLD	rs75648078	0.004	21.185	23.514
Rhodococcus_MASLD	rs79408227	0.004	23.867	23.514
Staphylococcus aureus_MASLD	rs12296330	0.004	21.868	23.414
Staphylococcus aureus_MASLD	rs150496956	0.004	21.67	23.414
Staphylococcus aureus_MASLD	rs1670340	0.004	23.397	23.414
Staphylococcus aureus_MASLD	rs55867611	0.004	23.447	23.414
Staphylococcus aureus_MASLD	rs6833195	0.004	21.09	23.414
Staphylococcus aureus_MASLD	rs73202240	0.004	22.844	23.414
Staphylococcus aureus_MASLD	rs74557139	0.005	31.45	23.414
Staphylococcus aureus_MASLD	rs77365752	0.004	21.386	23.414
Staphylococcus aureus_MASLD	rs7788178	0.004	23.572	23.414
Tannerellaceae_MASLD	rs10180730	0.004	21.401	23.337
Tannerellaceae_MASLD	rs10822361	0.004	21.055	23.337
Tannerellaceae_MASLD	rs11924583	0.004	24.484	23.337
Tannerellaceae_MASLD	rs12077014	0.004	21.324	23.337
Tannerellaceae_MASLD	rs12673506	0.005	31.29	23.337
Tannerellaceae_MASLD	rs16880908	0.004	21.968	23.337
Tannerellaceae_MASLD	rs17524995	0.004	23.554	23.337
Tannerellaceae_MASLD	rs1877593	0.004	21.963	23.337
Tannerellaceae_MASLD	rs2425862	0.004	21.528	23.337
Tannerellaceae_MASLD	rs2655609	0.004	21.457	23.337
Tannerellaceae_MASLD	rs34221271	0.004	22.244	23.337
Tannerellaceae_MASLD	rs4843380	0.004	21.744	23.337
Tannerellaceae_MASLD	rs60892486	0.004	24.318	23.337
Tannerellaceae_MASLD	rs7073854	0.004	21.989	23.337
Tannerellaceae_MASLD	rs7580217	0.005	29.737	23.337
UNC496MF_MASLD_	rs114698862	0.005	28.873	23.792
UNC496MF_MASLD_	rs13255030	0.004	21.707	23.792
UNC496MF_MASLD_	rs146721298	0.004	25.541	23.792
UNC496MF_MASLD_	rs2465153	0.004	22.364	23.792
UNC496MF_MASLD_	rs2842124	0.004	25.328	23.792
UNC496MF_MASLD_	rs2859246	0.005	28.768	23.792
UNC496MF_MASLD_	rs387455	0.004	22.742	23.792
UNC496MF_MASLD_	rs56160072	0.004	21.686	23.792
UNC496MF_MASLD_	rs56295230	0.004	20.963	23.792
UNC496MF_MASLD_	rs62495394	0.004	23.743	23.792
UNC496MF_MASLD	rs72679176	0.004	22.516	23.792
UNC496MF_MASLD	rs8192856	0.004	21.277	23.792

Supplementary Table s1.3 The R2, F and meanf of m6A.exposure_GM.outcome.

m6A.exposure_GM.outcome	SNP	R2	F	meanf
ALKBH3_GM	rs11037662	0.001	43.47	137.838
ALKBH3_GM	rs11037712	0.002	49.297	137.838
ALKBH3_GM	rs178517	0.001	37.882	137.838
ALKBH3_GM	rs2074038	0.002	69.148	137.838
ALKBH3_GM	rs34239319	0.008	237.265	137.838
ALKBH3_GM	rs35278338	0.002	47.917	137.838
ALKBH3_GM	rs4755217	0.018	578.573	137.838
ALKBH3_GM	rs61884011	0.001	39.153	137.838
ALKBH5_GM	rs1064629	0.011	157.202	100.521
ALKBH5_GM	rs11870660	0.003	38.279	100.521
ALKBH5_GM	rs138448950	0.003	34.46	100.521
ALKBH5_GM	rs2925138	0.021	305.952	100.521
ALKBH5_GM	rs35590625	0.004	33.503	100.521
ALKBH5_GM	rs55784499	0.004	47.904	100.521
ALKBH5_GM	rs62072547	0.012	136.964	100.521
ALKBH5_GM	rs9895454	0.003	49.902	100.521
CBLL1_GM	rs117859243	0.002	51.53	117.243
CBLL1_GM	rs12705420	0.002	48.839	117.243
CBLL1_GM	rs1468435	0.006	188.045	117.243
CBLL1_GM	rs150942317	0.001	32.38	117.243
CBLL1_GM	rs73727206	0.002	52.066	117.243
CBLL1_GM	rs739518	0.012	368.068	117.243
CBLL1_GM	rs77888195	0.003	79.773	117.243
RBM15B_GM	rs114740115	0.005	123.889	127.475
RBM15B_GM	rs1370124	0.003	92.448	127.475
RBM15B_GM	rs17606778	0.001	34.239	127.475
RBM15B_GM	rs2311059	0.001	36.849	127.475
RBM15B_GM	rs62257549	0.014	443.722	127.475
RBM15B_GM	rs66793762	0.001	41.31	127.475
RBM15B_GM	rs78104031	0.004	119.869	127.475
