# Supplementary material for: Mediation Role of Gut Microbiota in the Causal Relationship Between m6A Regulatory Genes and Metabolic Dysfunction-Associated Steatotic Liver Disease: A Mendelian Randomization Study
Source: Biomedicines. 2026 Mar 11;14(3):630. doi: 10.3390/biomedicines14030630 (PMC13023808; doi:10.3390/biomedicines14030630)
Supplement: Supplementary file 1 [file biomedicines-14-00630-s001.zip › Supplementary Table S3_All results of MRanalysis (the Heterogeneity p-value, Pleiotropy p-value, MR-PRESSO p-value).pdf]

Supplementary Table s3.1 The Heterogeneity P-value, Pleiotropy P-value, MR-PRESSO P-value of MR analysis of m6A Regulatory Genes on MASLD

M6A_ MASLD	Number of SNPs	Heterogeneity *P*-value	Pleiotropy *P*-value	MR-PRESSO *P*-value
ALKBH1_ MASLD	14	0.135	0.245	0.172
ALKBH3_ MASLD	9	0.557	0.684	0.639
ALKBH5_ MASLD	8	0.261	0.853	0.336
CBLL1 _ MASLD	7	0.214	0.757	0.273
CEBPZ _ MASLD	33	0.591	0.653	0.612
FTO _ MASLD	11	0.324	0.107	0.381
KIAA1429_ MASLD	7	0.619	0.437	0.550
METTL5_ MASLD	7	0.897	0.339	0.826
METTL7A_ MASLD	18	0.021	0.914	0.034
METTL14_ MASLD	11	0.955	0.833	0.956
RBM15_ MASLD	5	0.686	0.837	0.747
RBM15B_ MASLD	8	0.753	0.164	0.785
SMAD3_ MASLD	8	0.375	0.440	0.463
WTAP_ MASLD	8	0.378	0.077	0.446
YTHDF3_ MASLD	7	0.994	0.970	0.989

Supplementary Table s3.2 The Heterogeneity P-value, Pleiotropy P-value, MR-PRESSO P-value of MR analysis of GM on MASLD

GM_ MASLD	Number of SNPs	Heterogeneity *P*-value	Pleiotropy *P*-value	MR-PRESSO *P*-value
An181_ MASLD	6	0.539	0.603	0.580
Bacillus U _ MASLD	9	0.978	0.540	0.978
Bacillus velezensis _ MASLD	6	0.621	0.886	0.706
Bifidobacterium adolescentis _ MASLD	11	0.617	0.381	0.673
Blautia A sp002159835 _ MASLD	8	0.845	0.962	0.862
CAG-145 sp00232000 _ MASLD	5	0.250	0.190	0.290
CAG-180 sp000432435 _ MASLD	7	0.979	0.769	0.976
Demequina _ MASLD	6	0.807	0.393	0.837
Erysipelatoclostridiaceae _ MASLD	7	0.575	0.278	0.632
Halarcobacter _ MASLD	10	0.978	0.744	0.982
Herbidospora_ MASLD	9	0.974	0.894	0.983
Lactococcus lactis _ MASLD	7	0.717	0.488	0.742
Methanobrevibacter B _ MASLD	9	0.549	0.321	0.561
Olsenella C_ MASLD	10	0.601	0.507	0.642
Parabacteroides_ MASLD	12	0.844	0.593	0.857
Rhodococcus_ MASLD	10	0.971	0.825	0.976
Staphylococcus aureus_ MASLD	9	0.290	0.384	0.314
Tannerellaceae_ MASLD	14	0.843	0.318	0.851
UNC496MF_ MASLD	12	0.560	0.265	0.586

Supplementary Table s3.3 The Heterogeneity P-value, Pleiotropy P-value, MR-PRESSO P-value of MR analysis of rGM on MASLD

rGM_ MASLD	Number of SNPs	Heterogeneity *P*-value	Pleiotropy *P*-value	MR-PRESSO *P*-value
An181_ MASLD	68	0.652	0.388	0.662
Bacillus U _ MASLD	68	0.268	0.528	0.284
Bacillus velezensis _ MASLD	68	0.737	0.239	0.767
Bifidobacterium adolescentis _ MASLD	68	0.725	0.450	0.729
Blautia A sp002159835 _ MASLD	68	0.203	0.653	0.209
CAG-145 sp00232000 _ MASLD	68	0.490	0.471	0.468
CAG-180 sp000432435 _ MASLD	68	0.209	0.568	0.246
Demequina _ MASLD	68	0.664	0.543	0.662
Erysipelatoclostridiaceae _ MASLD	68	0.411	0.264	0.424
Halarcobacter _ MASLD	68	0.272	0.538	0.283
Herbidospora_ MASLD	68	0.827	0.140	0.815
Lactococcus lactis _ MASLD	68	0.028	0.790	0.031
Methanobrevibacter B _ MASLD	68	0.048	0.880	0.039
Olsenella C_ MASLD	68	0.140	0.240	0.145
Parabacteroides_ MASLD	68	0.438	0.582	0.453
Rhodococcus_ MASLD	68	0.786	0.929	0.805
Staphylococcus aureus_ MASLD	68	0.128	0.831	0.139
Tannerellaceae_ MASLD	68	0.408	0.598	0.446
UNC496MF_ MASLD	68	0.327	0.696	0.364

Supplementary Table s3.4 The Heterogeneity P-value, Pleiotropy P-value, MR-PRESSO P-value of MR analysis of M6A on GM.

M6A_ GM	Number of SNPs	Heterogeneity *P*-value	Pleiotropy *P*-value	MR-PRESSO *P*-value
ALKBH3_Bacillus velezensis	8	0.503	0.315	0.641
ALKBH3_Demequina	8	0.615	0.439	0.684
ALKBH5_Methanobrevibacter	8	0.937	0.588	0.962
ALKBH5_Olsenella C	8	0.795	0.535	0.862
ALKBH5_Parabacteroides	8	0.068	0.634	0.152
ALKBH5_Tannerellaceae	8	0.051	0.797	0.096
CBLL1_Tannerellaceae	7	0.659	0.537	0.518
RBM15B_Olsenella C	7	0.733	0.636	0.766
RBM15B_Staphylococcus aureus	7	0.971	0.638	0.971
RBM15B_Tannerellaceae	7	0.880	0.989	0.887
